# Fine mapping-based multi-omics analysis interprets the gut-lung axis function of SGLT2 inhibitors

**DOI:** 10.3389/fcimb.2024.1447327

**Published:** 2024-09-10

**Authors:** Fengqin Yuan, Tianlong Zhang, Sixiang Jia, Jianqiang Zhao, Binbin Wan, Gang Liu

**Affiliations:** ^1^ Department of Infection Control, the Fourth Affiliated Hospital of School of Medicine, and International School of Medicine, International Institutes of Medicine, Zhejiang University, Yiwu, China; ^2^ Department of Critical Care Medicine, the Fourth Affiliated Hospital of School of Medicine, and International School of Medicine, International Institutes of Medicine, Zhejiang University, Yiwu, China; ^3^ Department of Cardiology, the Fourth Affiliated Hospital of School of Medicine, and International School of Medicine, International Institutes of Medicine, Zhejiang University, Yiwu, China; ^4^ Department of Immunization Planning, Yiwu Center for Disease Control and Prevention, Yiwu, Zhejiang, China

**Keywords:** sodium-glucose cotransporter 2 inhibition, gut microbiota, circulating metabolites, interstitial lung disease, pulmonary tuberculosis, pneumoconiosis, asthma, fine mapping

## Abstract

**Background:**

Currently, Sodium-glucose cotransporter 2 (SGLT2) inhibitors demonstrate additional effects beyond glucose control on the gut microbiota and circulating metabolites. The gut microbiota and metabolites have been found to be useful in elucidating potential biological mechanisms of pulmonary diseases. Therefore, our study aims to investigate the effects of gut microbiota and metabolites mediating SGLT2 inhibition in 10 pulmonary diseases through Mendelian randomization (MR) research.

**Methods:**

We conducted a two-sample, two-step MR study to assess the association between SGLT2 inhibition and 10 pulmonary diseases and to investigate the mediating effects of gut microbiota and metabolite. Gene-fine mapping and annotation of mediators by FUMA and Magma analyses were performed, and causal associations of mapped genes with diseases were assessed by muti-omics MR analyses. Possible side effects of SGLT2 inhibition were assessed by PheWAS analysis.

**Results:**

SGLT2 inhibition was linked to a reduced risk of T2DM, Interstitial lung disease (ILD), Pneumoconiosis, Pulmonary tuberculosis, and Asthma(OR=0.457, 0.054, 0.002, 0.280, 0.706). The family Enterobacteriaceae and order Enterobacteriales were associated with SGLT2 inhibition and ILD(95% CI:0.079–0.138). The family Alcaligenaceae and X-12719 were linked to pneumoconiosis (95% CI: 0.042–0.120, 0.050–0.099). The genus Phascolarctobacterium was connected to pulmonary tuberculosis (95% CI: 0.236–0.703).The degree of unsaturation (Fatty Acids), ratio of docosahexaenoic acid to total fatty acids, and 4-androsten-3beta,17beta-diol disulfate 2, were associated with asthma(95% CI: 0.042–0.119, 0.039–0.101, 0.181–0.473). Furthermore, Fuma and Magma analyses identified target genes for the four diseases, and proteomic MR analysis revealed six overlapping target genes in asthma. PheWAS analysis also highlighted potential side effects of SGLT2 inhibition.

**Conclusions:**

This comprehensive study strongly supports a multi-omics association between SGLT2 inhibition and reduced risk of interstitial lung disease, tuberculosis, pneumoconiosis, and asthma. Four identified gut microbiota, four metabolites, sixteen metabolic pathways, and six target genes appear to play a potential role in this association. The results of the comprehensive phenome-wide association analysis also identified the full effect of SGLT2 inhibitors.

## 1 Introduction

Pulmonary diseases are intricately linked to human life and lead to significant public health challenges on a global scale due to their high incidence or mortality rates (Zhou et al., 2019). In recent years, with the emergence of gut microbiota, metabolomics, and proteomics, this area is receiving increasing attention. The discovery of the gut-lung axis suggests the potential manipulative role of the gut microbiome(gene) in treating pulmonary diseases (Budden et al., 2017). Metabolomics, through the study of metabolites, reveals potential biomarkers that can pave the way for disease prevention (Qiu et al., 2023). Metabolites, serving as the end products or intermediate compounds of metabolism, fulfill essential functions in the human body. The exploration of changes in intermediate metabolites or metabolic pathways provides a profound understanding of the progression of diseases (Johnson et al., 2016). Changes in blood proteins more finely reflect changes in the functioning of the organism. Thus, co-alterations in the gut microbiota, metabolites, and their proteins may play a key role in the etiologic formation of lung-related diseases and the identification of key therapeutic targets.

Sodium-glucose cotransporter 2 (SGLT2) inhibitors constitute a class of oral antidiabetic drugs, which includes dapagliflozin, empagliflozin, and others (Zannad et al., 2020). These inhibitors have been found to affect glycemic control, as well as potentially altering levels of gut microbiota and blood metabolites (particularly amino acids, ketones, and lipids), which can affect disease progression (Kappel et al., 2017; Cowie and Fisher, 2020; Herat et al., 2020; Szekeres et al., 2021). This finding may play a crucial role in improving pulmonary diseases.

Exploring the impact of SGLT2 inhibitors on pulmonary diseases faces significant challenges. Recently, Mendelian randomization (MR) has gained prominence as a widely used research method to assess the causal effects between exposure and outcomes, while minimizing biases arising from confounding factors or reverse causation (Kintu et al., 2023). MR analysis utilizes individual genetic variations as instrumental variables (IVs), simulating a randomized controlled trial (Georgakis and Gill, 2021). We employed this method, leveraging extensive Genome-Wide Association Studies (GWAS) and identified single nucleotide polymorphisms (SNPs) associated with SGLT2 inhibitors, to establish the causal relationship between SGLT2 inhibitors and pulmonary diseases.

The previous studies have applied MR analysis to assess the relationship between SGLT2 inhibitors and atrial fibrillation, coronary artery disease, and fractures, involving circulating metabolites as intermediaries (Xu et al., 2022; Dai et al., 2023; Li et al., 2023). Therefore, to gain a more comprehensive understanding of the association between SGLT2 and pulmonary diseases, building upon these studies, we hypothesize that gut microbiota and circulating metabolites may mediate the impact of SGLT2 inhibition on pulmonary diseases. Therefore, the objective of this article is to implement a two-sample MR approach to: (1) assess the causal effects of SGLT2 inhibitors and pulmonary diseases; (2) investigated the potential causal effects of SGLT2 inhibitors on gut microbiota and circulating metabolites; (3) conducted a two-step MR study to determine the mediating effects of gut microbiota and circulating metabolites on the relationship between SGLT2 inhibitors and pulmonary diseases; (4) analyzed relevant metabolites to gain insights into potential metabolic pathways underlying the association between SGLT2 and pulmonary diseases; (5) fine mapping based on instrumental variables of mediators and proteomic MR analysis to obtain effector genes; and (6) phenome-wide association analyses to determine potential side effects of SGLT2 inhibitors.

## 2 Materials and methods

### 2.1 Study design

**Figure 1:**
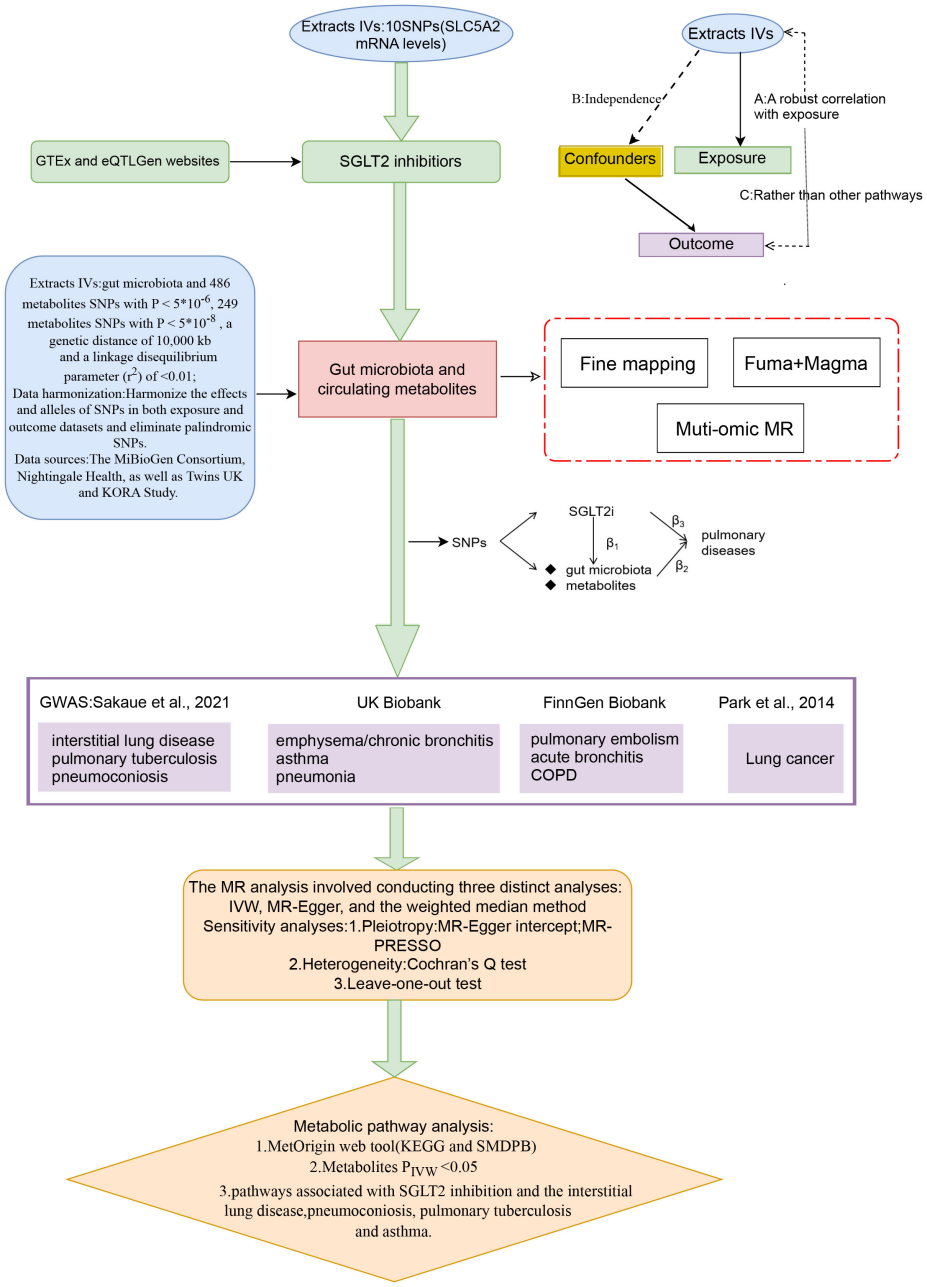
A schematic overview of the study design. We conducted a two-sample, two-step MR study to assess the association between SGLT2 inhibition and 10 pulmonary diseases and to investigate the mediating effects of gut microbiota and metabolite. Gene-fine mapping and annotation of mediators by FUMA and Magma analyses were performed, and causal associations of mapped genes with diseases were assessed by muti-omics MR analyses. IVs, instrumental variables; IVW, inverse-variance weighted; MR, Mendelian randomization; SNPs, single nucleotide polymorphisms.

**Figure 1** presents a schematic overview of the study design. The MR design should meet three necessary assumptions (**Figure 1**): (A) The genetic variants selected as instrumental variables (IVs) should demonstrate a robust correlation with the exposure (SGLT2 inhibitors, gut microbiota, metabolites); (B) The genetic instruments should exhibit no correlation with the occurrence of pulmonary diseases and should remain independent of potential confounding factors; (C) The genetic variant should show a distinct association with pulmonary diseases, mediated specifically through exposure rather than other pathways.

### 2.2 IVs for SGLT2 inhibitors

IVs for SGLT2 inhibitors were obtained by the study methodology reported in a previous article (Li et al., 2023). In brief, SNP information concerning SLC5A2 mRNA levels was obtained from blood or whole tissue data available on the Genotype-Tissue Expression (GTEx) (2020) and eQTLGenwebsites (Võsa et al., 2021). The SNPs were evaluated and screened for variants significantly associated with glycated hemoglobin (HbA1c) levels (glucose-lowering target) on the basis of r^2^ < 0.8 and 250 kb (P< 1×10^-4^). Finally, shared variants between the two were confirmed by co-localization analysis.

### 2.3 Data sources on the gut microbiota and circulating metabolites

The MiBioGen Consortium provided comprehensive summary statistics on the genetic impact on the human gut microbiota, including genome-wide genotyping data from 14,363 individuals of European ancestry (Kurilshikov et al., 2021). A total of 211 taxonomic groups (16 orders, 35 families, 131 genera, 20 classes, 9 phyla) were included in the relevant analysis of this study. To incorporate a more comprehensive set of metabolites, we selected data from two sources. One dataset comprised 249 circulating metabolites from 121,000 participants of European ancestry, generated by Nightingale Health, primarily covering lipids and lipoprotein particles (81%) (Ritchie et al., 2023). The second dataset involved 486 metabolites from 7,824 participants of European ancestry in the TwinsUK and KORA studies, encompassing eight categories of metabolites: Amino acid, Lipid, Carbohydrate, Nucleotide, Energy, Cofactors and vitamins, Peptide, and Xenobiotics (Shin et al., 2014). For overlapping metabolites from two data sources, we chose to exclude them if the direction of their impact on the outcomes of pulmonary diseases was inconsistent.

### 2.4 IVs selection for gut microbiota and circulating metabolites

In the analysis of 249 metabolites, SNPs with p-values below the genome-wide significance threshold (5 × 10^-8^) were selected as IVs. In the analysis of 211 gut microbiota and 486 blood metabolites, to enhance sensitivity to IVs and obtain more comprehensive results, SNPs with p-values below the genome-wide significance threshold (5 × 10^-6^) were chosen as IVs. Subsequently, all IVs underwent linkage disequilibrium (LD) clumping (r^2^ = 0.01; distance = 10,000 kb) to alleviate the impact of correlated SNPs. Additionally, we used the PhenoScanner version to prevent the selected SNP from showing significant pleiotropic associations. Furthermore, we calculated the F-statistic (R^2^ (N–2)/(1–R^2^)), assessing the strength of each instrument, where R^2^ represents the proportion of variance explained by the genetic instrument, and N is the effective sample size (Kwok et al., 2020). Finally, we excluded palindromic SNPs from our study.

### 2.5 IVs selection for pQTL

pQTL data for blood proteins were obtained from a study based on the Icelandic (n = 35559) population (Ferkingstad et al., 2021) and were analyzed in the MR analysis using the same SNP screening conditions as previously (P < 5 × 10^-8^).

### 2.6 Data sources on the pulmonary diseases

Ten pulmonary diseases are categorized according to clinical criteria outlined by the World Health Organization (WHO) and the tenth edition of the International Classification of Diseases (ICD-10). Data summaries for interstitial lung disease, pulmonary tuberculosis, and pneumoconiosis were extracted from publicly accessible datasets as provided by the GWAS conducted by Sakaue et al (Sakaue et al., 2021). For asthma, pneumonia, and emphysema/chronic bronchitis outcomes, we utilized data from the UK Biobank, a large cohort of UK adult volunteers, to provide detailed information (Bycroft et al., 2018). Summary-level data for pulmonary embolism, acute bronchitis, and chronic obstructive pulmonary disease (COPD) were generated from the FinnGen Biobank (Kurki et al., 2023). Lung cancer was sourced from the Transdisciplinary Research into Cancer of the Lung consortium (Park et al., 2014). All studies within these consortia obtained approval from local research ethics committees and institutional review boards, and participants in each study provided written informed consent. **Table 1** outlines the characteristics of the summarized datasets for the ten pulmonary diseases.

**Table 1:** The characteristics of the ten pulmonary diseases.

Pulmonary diseases	Ethnicity	Sample size	Cases	Control
Interstitial lung disease	European	469,827	2,267	467,560
Pulmonary tuberculosis	European	477,386	895	476,491
Pneumoconiosis	European	479,040	433	478,607
Asthma	European	408,442	56,167	352,255
Pulmonary embolism	European	218,413	4,185	214,228
Acute bronchitis	European	216,027	7,338	208,689
Chronic obstructive pulmonary disease	European	193,318	6,595	186,723
Pneumonia	European	486,484	22,567	463,917
Emphysema/chronic bronchitis	European	462,013	7,844	454,169
Lung cancer	European	40,453	23,848	16,605

### 2.7 MR statistical analysis

A two-sample MR analysis was employed to assess the causal relationship between SGLT2 inhibition and ten pulmonary diseases as well as T2DM. A two-step MR was performed to estimate the mediating effect of gut microbiota and circulating metabolites on the association between SGLT2 inhibition and positive pulmonary diseases. The first step of the two-step MR involved assessing the impact of SGLT2 inhibition on gut microbiota and circulating metabolites (beta1). In the second step, we evaluated the influence of gut microbiota and metabolites significantly associated with SGLT2 inhibition on the positive pulmonary diseases (beta2). The proportion mediated by gut microbiota and metabolites in the association between SGLT2 inhibition and positive pulmonary diseases was calculated as the product of beta1 and beta2 divided by the total effect of SGLT2 inhibition on positive pulmonary diseases (beta 3). The 95% confidence interval for the mediation proportion was calculated using the product of coefficients method.

The primary analysis employed the IVW method to derive the final effect estimate. To ensure the accuracy of the results, additional sensitivity analyses were performed, including the MR-Egger method, weighted median analysis, and MR pleiotropy residual sum and outlier (MR-PRESSO) test. The MR Egger method, known for accommodating directional horizontal pleiotropic effects, addresses the possibility of SNP effects on target outcomes through alternative biological pathways independent of the investigated exposure (Wu et al., 2020). SNPs were assessed using a weighted median approach, considering precision relative to magnitude, with the median determining the overall MR estimate, and standard errors estimated through bootstrapping (Mohus et al., 2022). The MR-PRESSO test identified potential horizontal pleiotropy, and its impact was addressed by excluding outliers from the analysis (Verbanck et al., 2018). Leave-one-out analyses were conducted to assess pleiotropy associated with individual SNPs. Heterogeneity and outliers were examined using I^2^ and Cochran Q-derived P-values in the IVW and MR-Egger analyses.

In this study, a p-value less than 0.05 was considered nominally associated. When assessing the impact of SGLT2 inhibition on 10 pulmonary diseases, we employed Bonferroni correction (Sedgwick, 2014) to rigorously control for false positives across multiple tests. Additionally, for the associations of microbiota and metabolites with pulmonary diseases, we applied a slightly more lenient False Discovery Rate (FDR) correction (Storey and Tibshirani, 2003) to control for false positives across multiple tests. To identify additional microbiota and metabolites that may potentially be associated, we expanded the FDR-corrected p-value threshold to 0.2.

Statistical analyses were executed with R software version 4.2.3, and the MR analyses were carried out using the TwoSampleMR package along with the MRPRESSO package.

### 2.8 Fine mapping to identify target gene affecting disease

We used FUMA (https://fuma.ctglab.nl/) for fine mapping of instrumental variables for metabolites and gut microbiota with mediating effects, using a maximum P-value of 1× 10^-5^ for lead SNPs for annotation (Watanabe et al., 2017).

### 2.9 Magma analysis

The genes obtained by fuma are put into the “GENE2FUNC function” to annotate the genes according to the biological background. GTEX V8 was used as the background gene set.

### 2.10 Muti-omic analysis

Two-sample MR analysis was performed to assess the causal relationship between pQTL of finely mapped acquired genes and lung disease. P-value less than 0.05 was considered nominally associated.

### 2.11 PheWAS analysis

We conducted a comprehensive whole-phenotype MR analysis to explore the potential side effects associated with SGLT2 inhibitors. In this study, we utilized gene expression data as the exposure variable and disease summary statistics from the UK Biobank cohort, encompassing a substantial sample size of up to 408,961 individuals, as the outcome measure. To ensure robustness in our phenotype-MR analysis, we meticulously selected 783 distinct traits, each with a substantial case count exceeding 500, thereby enhancing the reliability and generalizability of our findings.

### 2.12 Metabolic pathway analysis

We conducted an analysis of metabolic pathways using the network-based MetOrigin (http://metorigin.met-bioinformatics.cn/) (Yu et al., 2022) for functional enrichment and pathway exploration. This approach aimed to identify potential connections between metabolic pathways, metabolite groups, or biological processes and the impact of SGLT2 inhibitors on pulmonary diseases. The significance threshold for the pathway analysis was set at 0.05. Additionally, our study only focused on analyzing metabolites identified by the IVW method with associations surpassing the recommended threshold (P < 0.05).

## 3 Results

### 3.1 Selection of IVs

Ten independent IVs were chosen as genetic instruments for SGLT2 inhibition (**Supplementary Table S1**). The analysis involved a range of 3 to 12 selected IVs for gut microbiota (**Supplementary Table S1**) and 3 to 159 IVs for metabolites (**Supplementary Tables S2**, **S3**). It is crucial to note that the F statistics for all SNPs surpassed the threshold of 10, suggesting a negligible probability of encountering weak instrument bias.

### 3.2 Causal effects of SGLT2 inhibition on 10 pulmonary diseases and T2DM

**Figure 2:**
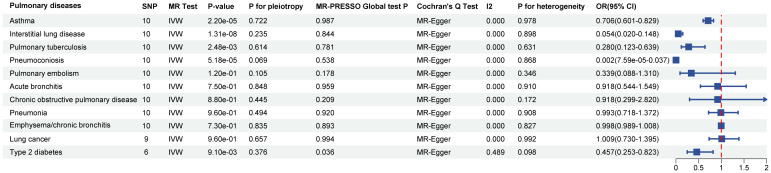
Causal effects of SGLT2 inhibition on 10 pulmonary diseases and T2DM. Forest plot showing the Inverse Variance-Weighted method for determining causal associations between SGLT2 inhibitors and ten prior lung diseases. MR-ivw analyses with P-value < 0.05 were determined to be positive results, P for pleiotropy > 0.05 showed that MR analyses were not pleiotropic, and P for heterogeneity > 0.05 indicated that MR analyses were not heterogeneous. IVW, inverse-variance weighted; OR, odds ratio.

In the MR analysis, after Bonferroni correction (p<0.05/11), we observed that SGLT2 inhibition was associated with a lowered risk of Interstitial lung disease (OR: 0.054, 95% CI: 0.020–0.148, p = 1.31×10^-8^), Pulmonary tuberculosis (OR: 0.280, 95% CI: 0.123–0.639, p = 2.48×10^-3^), Pneumoconiosis(OR: 0.002, 95% CI: 7.58×10^-5^–0.037, p = 5.18×10^-5^), Asthma (OR: 0.706, 95% CI:0.601–0.829, p = 2.22×10^-5^) and T2DM (OR: 0.457, 95% CI: 0.253–0.823, p = 9.13×10^-3^) (**Supplementary Table S4**). The results of the sensitivity analysis did not indicate the presence of heterogeneity or directional pleiotropy (**Figure 2**).

### 3.3 Causal effects of SGLT2 inhibition on gut microbiota and metabolites

**Figure 3:**
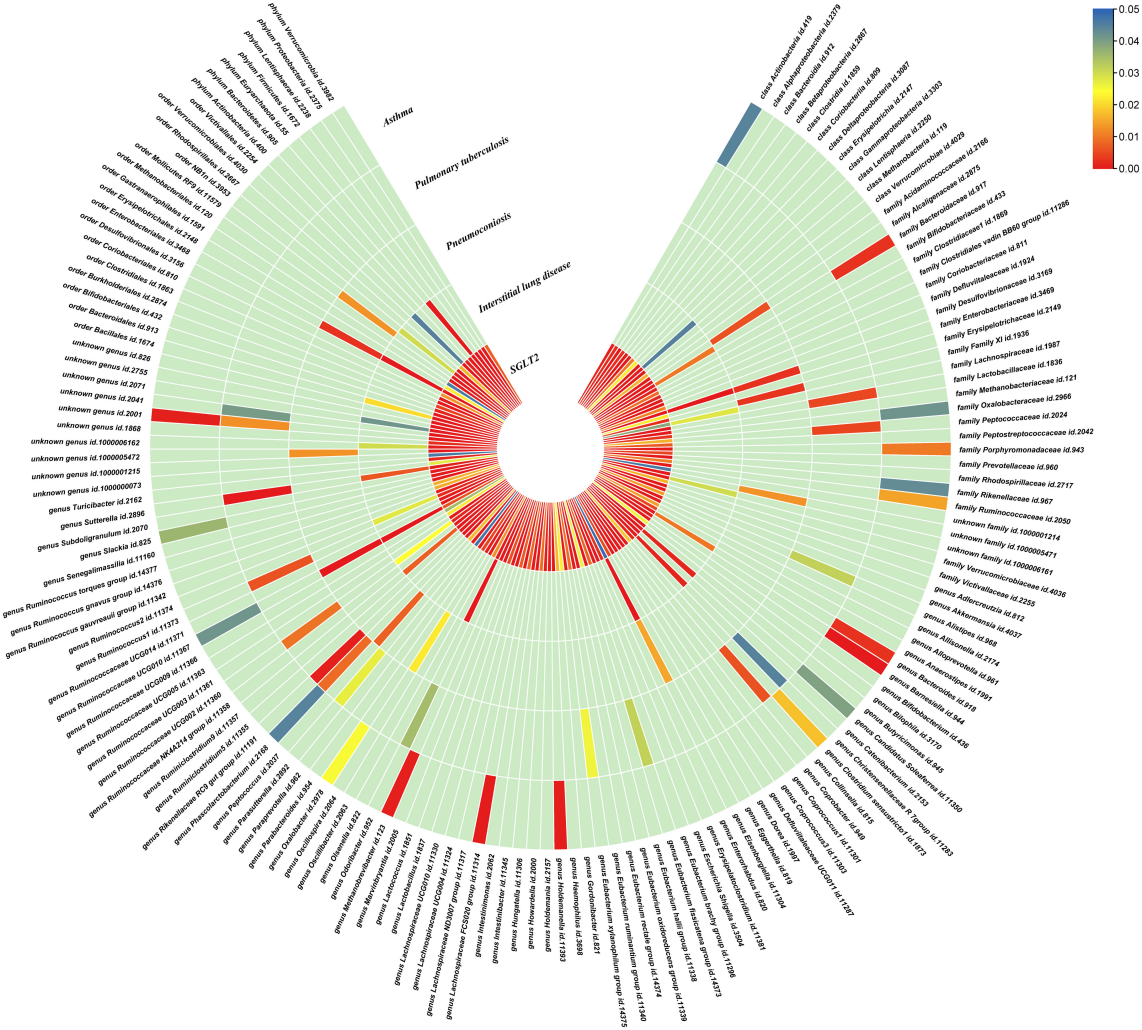
Causal effects of gut microbiota on interstitial lung disease, pulmonary tuberculosis, pneumoconiosis, and asthma. Circumferential thermograms showing causally linked gut microbiota in interstitial lung disease, tuberculosis, pneumoconiosis and asthma. The circular thermogram is divided into five layers representing the P-value results of MR analysis of SGLT2i, interstitial lung disease, pulmonary tuberculosis, pneumoconiosis, and asthma with gut microbiota. The change in color of each cell indicates the magnitude of the P-value. The redder the color of each cell, the smaller the P value; the bluer the color of each cell, the larger the value.

**Figure 4:**
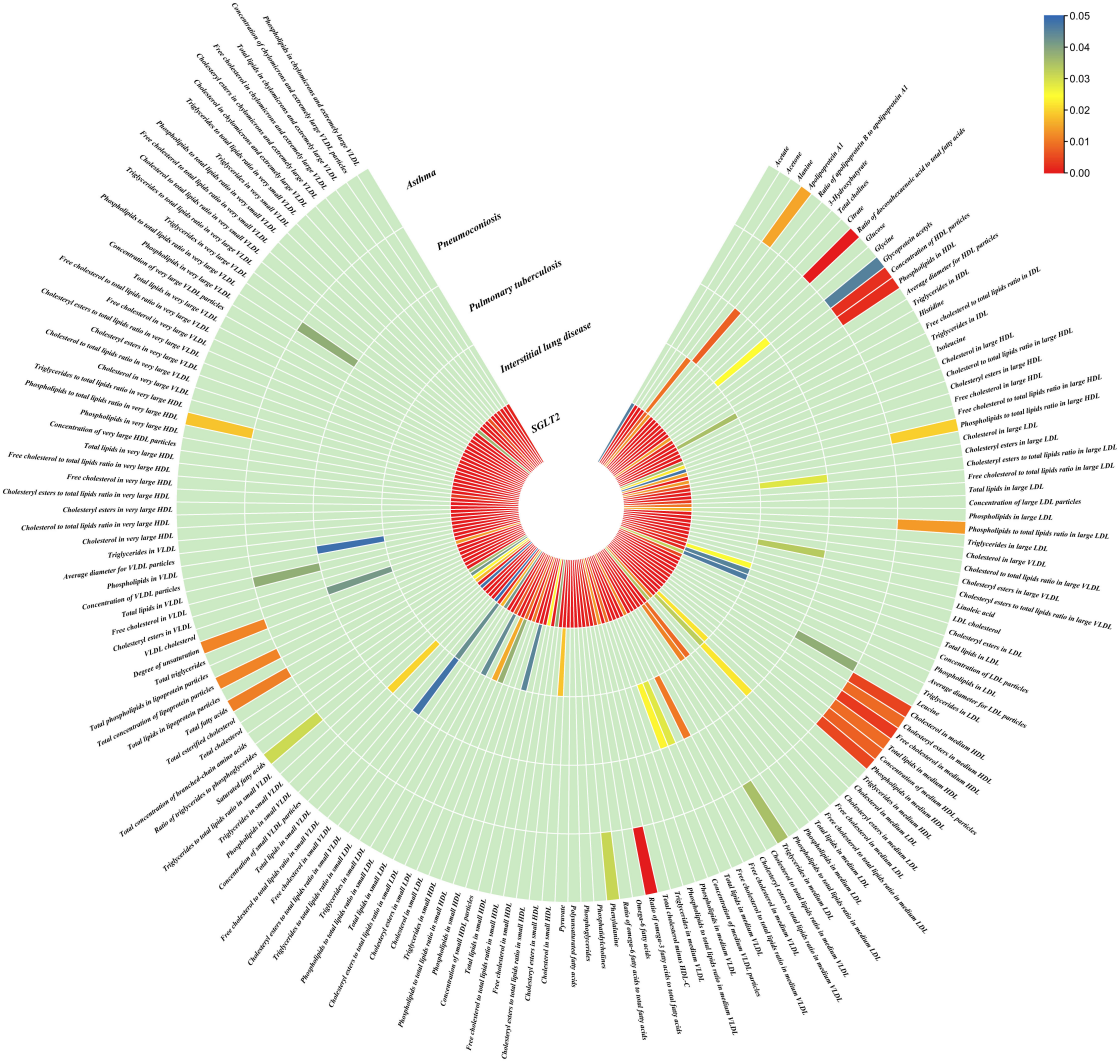
Causal effects of metabolites on interstitial lung disease, pulmonary tuberculosis, pneumoconiosis, and asthma. Circumferential thermograms showing causally linked metabolites (249 class) in interstitial lung disease, tuberculosis, pneumoconiosis and asthma. The circular thermogram is divided into five layers representing the P-value results of MR analysis of SGLT2i, interstitial lung disease, pulmonary tuberculosis, pneumoconiosis, and asthma with metabolites. The change in color of each cell indicates the magnitude of the P-value. The redder the color of each cell, the smaller the P value; the bluer the color of each cell, the larger the value.

**Figure 5:**
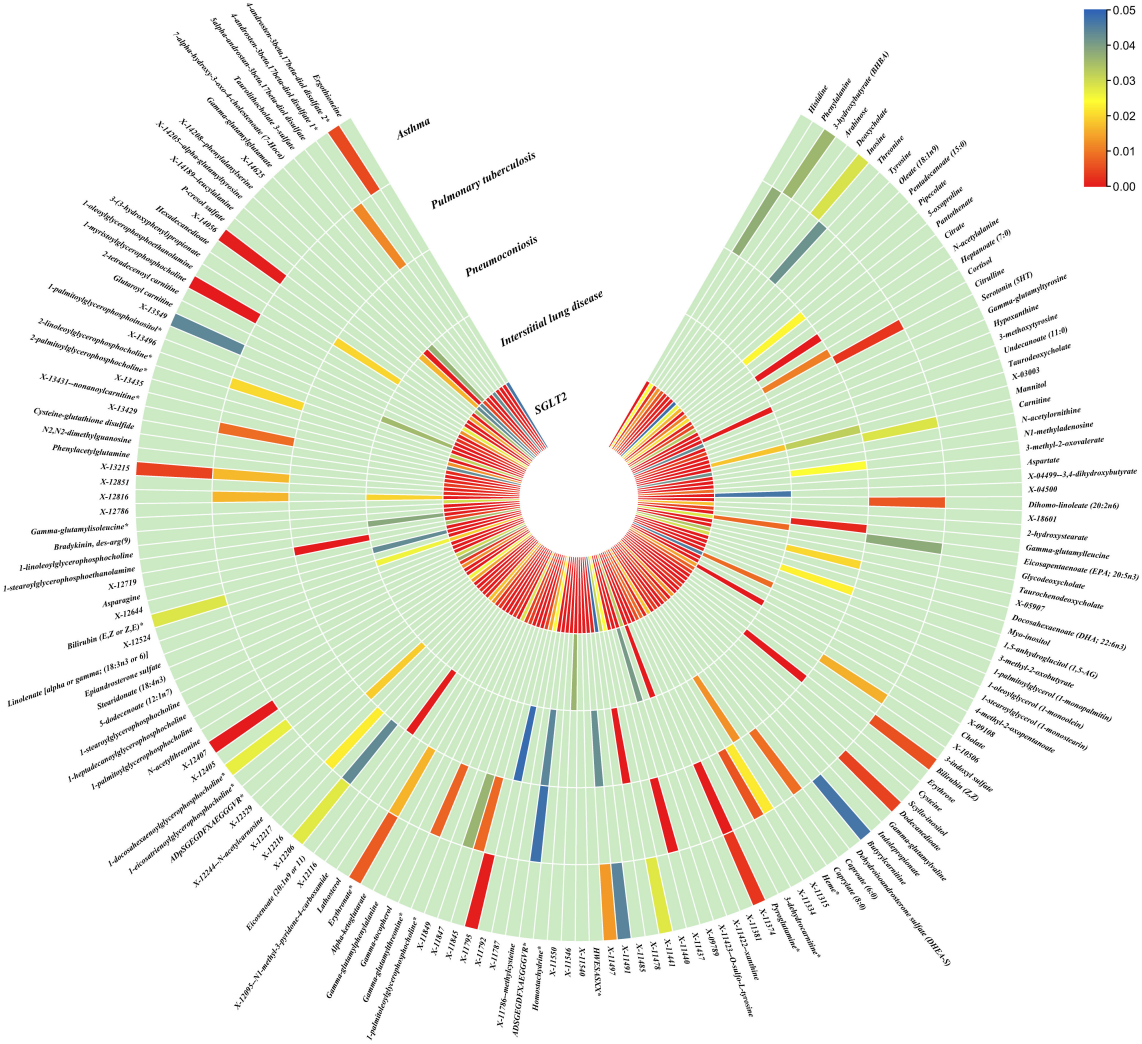
Causal effects of metabolites on interstitial lung disease, pulmonary tuberculosis, pneumoconiosis, and asthma. Circumferential thermograms showing causally linked metabolites (486 class) in interstitial lung disease, tuberculosis, pneumoconiosis and asthma. The circular thermogram is divided into five layers representing the P-value results of MR analysis of SGLT2i, interstitial lung disease, pulmonary tuberculosis, pneumoconiosis, and asthma with metabolites. The change in color of each cell indicates the magnitude of the P-value. The redder the color of each cell, the smaller the P value; the bluer the color of each cell, the larger the value.

The IVW method revealed 152 nominally significant associations (p < 0.05) between SGLT2 inhibition and gut microbiota, 173 nominal associations with 249 circulating metabolites, and 220 nominal associations with 486 metabolites (**Figures 3**–**5**; **Supplementary Table S4**).

### 3.4 Causal effects of gut microbiota and metabolites on interstitial lung disease, pulmonary tuberculosis, pneumoconiosis and asthma

We performed causal estimation for Interstitial Lung Disease, Pulmonary Tuberculosis, Pneumoconiosis, and Asthma using 152 nominally significant gut microbiota, 173 out of 249 circulating metabolites and 220 out of 486 metabolites, respectively (**Figures 3**–**5**). Among the 211 types of gut microbiota included in the analysis, a total of 22 gut microbiota (representing 1 order, 2 families, 7 genera, and 7 species) were found to have a causal relationship with Interstitial Lung Disease; 6 gut microbiota (representing 1 order, 4 families, 1 genera) were found to have a causal relationship with Pneumoconiosis; 8 gut microbiota (representing 2 families, 6 genera) were found to have a causal relationship with Pulmonary Tuberculosis; 8 gut microbiota (representing 2 families, 6 genera) were found to have a causal relationship with Asthma.

As for metabolites, X-11422–xanthine, X-09108, X-14208–phenylalanylserine, Gamma-glutamyltyrosine were highly correlated with interstitial lung disease;X-12719, Erythrose, N-acetylalanine, X-11478, X-12116, and Gamma-glutamylleucine were highly correlated with pneumoconiosis;X-11437 and X-11374 were highly correlated with pulmonary tuberculosis;17 out of 21 circulating metabolites, 10 out of 20 metabolites showed significant correlations with asthma.

The all results were as follows:

Interstitial lung disease: The IVW method revealed nominally significant associations (p < 0.05) between 22 gut microbiota,15 out of 173 circulating metabolites, and 17 out of 220 metabolites with interstitial lung disease. After FDR correction, we observed 12 gut microbiota, 0 out of 15 circulating metabolites, 4 out of 17 metabolites with significant causative correlations to interstitial lung disease. Among gut microbiota, the family Enterobacteriaceae id.3469 (OR: 1.577, 95% CI: 1.423–1.749, p = 4.93×10^-18^, FDR = 3.70×10^-16^), the family Bacteroidaceae id.917 (OR: 1.174, 95% CI: 1.039–1.327, p =0.01, FDR =0.125), the genus Eisenbergiella id.11304 (OR: 1.275, 95% CI: 1.158–1.404, p = 7.50×10^-7^, FDR = 3.75×10^-5^), the genus Ruminococcaceae UCG010 id.11367 (OR: 0.822, 95% CI: 0.760–0.890, p = 1.37×10^-6^, FDR = 5.14×10^-5^), the genus Odoribacter id.952 (OR: 1.463, 95% CI: 1.242–1.723, p = 5.05×10^-6^, FDR = 1.52×10^-4^), the genus Christensenellaceae R 7group id.11283 (OR: 0.523, 95% CI: 0.352–0.776, p = 1.28×10^-3^, FDR =0.026), the genus Candidatus Soleaferrea id.11350 (OR: 1.222, 95% CI: 1.080–1.382, p = 1.41×10^-3^, FDR =0.026), the genus Sutterella id.2896 (OR: 0.602, 95% CI: 0.602–0.921, p = 6.54×10^-3^, FDR =0.109), the genus Ruminiclostridium9 id.11357 (OR: 0.846, 95% CI: 0.748–0.956, p =7.45×10^-3^, FDR =0.112), the genus Bacteroides id.918(OR: 1.174, 95% CI: 1.039–1.327,p=0.01, FDR=0.125), the order Enterobacteriales id.3468 (OR: 1.577, 95% CI: 1.423–1.749, p = 4.93×10^-18^, FDR = 3.70×10^-16^), and the phylum Euryarchaeota id.55 (OR: 1.270, 95% CI: 1.115–1.446, p = 3.05×10^-4^, FDR = 7.62×10^-3^) were strongly associated with interstitial lung disease. As for metabolites, X-11422–xanthine(OR: 0.327, 95% CI: 0.219–0.489, p = 5.11×10^-8^, FDR =1.06×10^-5^), X-09108 (OR: 0.190, 95% CI: 0.084–0.428, p = 6.34×10^-5^, FDR = 6.56×10^-3^), X-14208–phenylalanylserine(OR: 0.650, 95% CI: 0.519–0.813, p = 1.70×10^-4^, FDR = 0.012), Gamma-glutamyltyrosine(OR: 4.357, 95% CI: 1.948–9.748, p = 3.4×10^-4^, FDR =0.018) were highly correlated with interstitial lung disease.

Pneumoconiosis: The IVW method revealed nominally significant 11 gut microbiota, 3 out of 173 circulating metabolites and 18 out of 220 metabolites. After FDR correction, we observed 6 gut microbiota, 0 out of 3 circulating metabolites, 6 out of 18 metabolites with significant correlations to pneumoconiosis. Among gut microbiota, the genus Ruminococcaceae UCG010 id.11367 (OR: 0.256, 95% CI: 0.205–0.318, p = 4.77×10^-34^, FDR =7.16×10^-32^), the genus Peptococcus id.2037 (OR: 0.577, 95% CI: 0.386–0.865, p = 7.66×10^-3^, FDR =0.191),the family Enterobacteriaceae id.3469 (OR: 0.263, 95% CI: 0.111–0.628, p = 2.60×10^-3^, FDR =0.112), the family Family XI id.1936 (OR: 1.597, 95% CI: 1.172–2.175, p = 3.00×10^-3^, FDR =0.112), the family Alcaligenaceae id.2875 (OR: 0.568, 95% CI: 0.379–0.850, p = 5.95×10^-3^, FDR =0.179), and the order Enterobacteriales id.3468 (OR: 0.263, 95% CI: 0.111–0.628, p = 2.60×10^-3^, FDR =0.112) were associated with pneumoconiosis. As for metabolites, X-12719 (OR: 3.793, 95% CI: 3.061–4.702, p = 4.25×10^-34^, FDR =8.79×10^-32^), Erythrose(OR: 8.096, 95% CI: 3.470–18.885, p = 1.30×10^-6^, FDR =1.35×10^-4^), N-acetylalanine (OR: 8.096, 95% CI: 3.470–18.885, p = 2.89×10^-5^, FDR =1.20×10^-3^), X-11478 (OR: 0.410, 95% CI: 0.268–0.629, p = 4.27×10^-5^, FDR =2.21×10^-3^), X-12116 (OR: 0.126, 95% CI: 0.041–0.389, p = 3.18×10^-4^, FDR =0.013), and Gamma-glutamylleucine (OR: 41.849, 95% CI: 4.207–416.246, p = 1.44×10^-3^, FDR =0.049) were highly correlated with pneumoconiosis.

Pulmonary Tuberculosis: The nominally significant associations included 16 gut microbiota, 12 out of 173 circulating metabolites, 24 out of 220 metabolites. After FDR correction, we observed 8 gut microbiota, 0 out of 12 circulating metabolites, 2 out of 24 metabolites with significant correlations to pneumoconiosis. Among gut microbiota, the genus Sutterella id.2896 (OR: 0.666, 95% CI: 0.609–0.728, p = 7.23×10^-19^, FDR =1.08×10^-16^), the genus Rikenellaceae RC9 gut group id.11191(OR: 0.885, 95% CI: 0.826–0.949, p = 5.59×10^-4^, FDR =0.042), the genus Collinsella id.815 (OR: 1.353, 95% CI: 1.090–1.679, p = 6.07×10^-3^, FDR =0.152), genus Ruminococcus1 id.11373 (OR: 0.798, 95% CI: 0.679–0.937, p = 6.03×10^-3^, FDR =0.152), the genus Phascolarctobacterium id.2168(OR: 0.714, 95% CI: 0.555–0.918, p = 8.62×10^-3^, FDR =0.180), the genus Ruminococcaceae UCG002 id.11360 (OR: 0.842, 95% CI: 0.739–0.959, p = 9.58×10^-3^, FDR =0.180), the family Lactobacillaceae id.1836 (OR: 0.967, 95% CI: 0.945–0.990, p = 4.87×10^-3^, FDR =0.152), and the family Peptococcaceae id.2024 (OR: 0.786, 95% CI: 0.665–0.929, p = 4.73×10^-3^, FDR =0.152) were associated with pulmonary tuberculosis. As for metabolites, X-11437 (OR: 1.064, 95% CI: 1.036–1.094, p = 8.09×10^-6^, FDR =1.68×10^-3^), and X-11374 (OR: 0.541, 95% CI: 0.390–0.752, p = 2.55×10^-4^, FDR =0.026) were highly correlated with pulmonary tuberculosis.

Asthma: The nominally significant associations included 18 gut microbiota, 21 out of 173 circulating metabolites, 20 out of 220 metabolites. After FDR correction, we observed 8 gut microbiota, 17 out of 21 circulating metabolites, 10 out of 20 metabolites showed significant correlations with asthma. Among gut microbiota, the genus Barnesiella id.944(OR: 1.072, 95% CI: 1.036–1.110, p = 7.10×10^-5^,FDR=0.011),the genus Holdemanella id.11393(OR: 0.951, 95% CI: 0.925–0.978, p = 4.47×10^-4^,FDR=0.016), the genus Lachnospiraceae FCS020 group id.11314 (OR: 0.934, 95% CI: 0.900–0.970, p = 4.08×10^-4^,FDR=0.016), the genus Methanobrevibacter id.123 (OR: 0.979, 95% CI: 0.968–0.991, p = 5.42×10^-4^,FDR=0.016), the unknown genus id.2001 (OR: 0.944, 95% CI: 0.913–0.976, p = 6.64×10^-4^,FDR=0.017), the genus Bacteroides id.918(OR: 1.135, 95% CI: 1.045–1.233, p = 2.63×10^-3^,FDR=0.049), the family Bacteroidaceae id.917 (OR: 1.135, 95% CI: 1.045–1.233, p = 2.63×10^-3^,FDR=0.049), and the family Porphyromonadaceae id.943(OR: 0.850, 95% CI: 0.751–0.962, p = 0.01, FDR=0.169) were highly associated with asthma. As for metabolites, Degree of unsaturation (Fatty Acids) (OR: 1.109, 95% CI: 1.066–1.154, p = 3.19×10^-7^, FDR =5.53×10^-5^), Ratio of docosahexaenoic acid to total fatty acids (OR: 1.121, 95% CI: 1.071–1.173, p = 8.36×10^-7^,FDR =7.23×10^-5^), Ratio of omega-3 fatty acids to total fatty acids(OR: 1.079, 95% CI: 1.038–1.122, p = 1.31×10^-4^,FDR =7.58×10^-3^), Phospholipids in HDL(OR: 0.947, 95% CI: 0.915–0.980, p = 1.95×10^-3^,FDR = 0.078), Concentration of HDL particles (OR: 0.933, 95% CI: 0.892–0.975, p = 2.26×10^-3^,FDR = 0.078), Concentration of medium HDL particles (OR: 0.952, 95% CI: 0.919–0.987, p = 7.73×10^-3^,FDR = 0.138), Cholesteryl esters in medium HD(OR: 0.953, 95% CI: 0.919–0.988, p = 8.61×10^-3^,FDR = 0.138), Total lipids in medium HDL(OR: 0.952, 95% CI: 0.918–0.988, p = 8.78×10^-3^,FDR = 0.138), Total fatty acids(OR: 0.945, 95% CI: 0.905–0.987, p = 0.011,FDR = 0.152), Total concentration of lipoprotein particles(OR: 0.945, 95% CI: 0.905–0.987, p = 0.011,FDR = 0.152), Phospholipids to total lipids ratio in large LDL(OR: 1.048, 95% CI: 1.010–1.088, p = 0.014,FDR = 0.171), Apolipoprotein A1(OR: 0.951, 95% CI: 0.914–0.990, p = 0.015,FDR = 0.173), Phospholipids to total lipids ratio in large HDL(OR: 1.051, 95% CI: 1.008–0.990, p = 1.095,FDR = 0.199), X-12407(OR: 0.920, 95% CI: 0.897–0.944, p = 1.36×10^-10^,FDR =2.84×10^-8^), 1-myristoylglycerophosphocholine(OR: 1.113, 95% CI: 1.061–1.169, p = 1.45×10^-5^,FDR =1.50×10^-3^), X-14056 (OR: 1.354, 95% CI: 1.142–1.604, p = 4.70×10^-4^, FDR =0.025), X-11795(OR: 1.696, 95% CI: 1.260–2.282, p = 4.89×10^-4^, FDR =0.025), X-11374(OR: 1.171, 95% CI: 1.055–1.298, p = 2.89×10^-3^, FDR =0.120), X-13215(OR: 0.773, 95% CI: 0.647–0.922, p = 4.25×10^-3^, FDR =0.130), Dodecanedioate (OR: 1.218, 95% CI: 1.063–1.395, p = 4.45×10^-3^, FDR =0.130), 4-androsten-3beta,17beta-diol disulfate 2* (OR: 0.758, 95% CI: 0.624–0.920, p = 5.01×10^-3^, FDR =0.130), Bilirubin (Z,Z) (OR: 0.938, 95% CI: 0.896–0.982, p = 5.83×10^-3^, FDR =0.135), and Erythronate*(OR: 0.666, 95% CI: 0.495–0.895, p = 7.01×10^-3^, FDR =0.146) were highly associated with asthma.

In relation to asthma, the Ratio of docosahexaenoic acid to total fatty acids and the Degree of unsaturation exhibited evidence of heterogeneity in Cochran’s Q test. MR-PRESSO identified outliers, providing corrected values after observing anomalies. The remaining results showed no evidence of heterogeneity in Cochran’s Q test, and MR-PRESSO did not detect any significant horizontal pleiotropy. (**Supplementary Tables S1**–**3**).

### 3.5 Mediation MR

After screening for potential intermediate factors, we identified a total of 4 gut microbiota and 4 metabolites that met our selection criteria.

Interstitial lung disease: We identified an indirect effect of SGLT2 inhibition on interstitial lung disease through the family Enterobacteriaceae id.3469 (OR: 0.499, 95% CI: 0.396–0.629, P = 3.71×10^-9^), and the order Enterobacteriales id.3468(OR: 0.499, 95% CI: 0.396–0.629, P = 3.71×10^-9^), with a mediated proportion of 10.87% (95% CI: 0.079–0.138, P = 2.08×10^-4^) and 10.87% (95% CI: 0.079–0.138, P = 2.08×10^-4^) of the total effect.

Pneumoconiosis: We identified an indirect effect of SGLT2 inhibition on pneumoconiosis through the family Alcaligenaceae id.2875 (OR: 0.401, 95% CI: 0.249–0.646, P = 1.71×10^-4^) and X-12719 (OR: 0.701, 95% CI: 0.503–0.975, P =0.035). The mediated proportion of the total effect was 8.10% (95% CI: 0.042–0.120, P =0.039) and 7.43% (95% CI: 0.050–0.099, P = 2.65×10^-3^), respectively.

Pulmonary tuberculosis: We found a mediated effect of SGLT2 inhibition on pulmonary tuberculosis through the involvement of the genus Phascolarctobacterium id.2168 (OR: 0.170, 95% CI: 0.123–0.236, P = 1.57×10^-26^). This mediation contributed significantly, accounting for 46.94% (95% CI: 0.236–0.703, P = 0.045) of the total effect.

Asthma: We identified an indirect effect of SGLT2 inhibition on asthma through the Degree of unsaturation (Fatty Acids) (OR: 0.762, 95% CI: 0.692–0.839, P = 2.75×10^-8^), Ratio of docosahexaenoic acid to total fatty acids (OR: 0.809, 95% CI: 0.734–0.891, P = 1.69×10^-5^),4-androsten-3beta,17beta-diol disulfate 2*(OR: 0.663, 95% CI: 0.540–0.814, P = 8.60×10^-5^),with a mediated proportion of 8.10% (95% CI: 0.042–0.119, P = 0.036), 6.96% (95% CI: 0.039–0.101, P = 0.025), 32.71% (95% CI: 0.181–0.473, P = 0.025), of the total effect.

### 3.6 Metabolic pathway analysis

**Figure 6:**
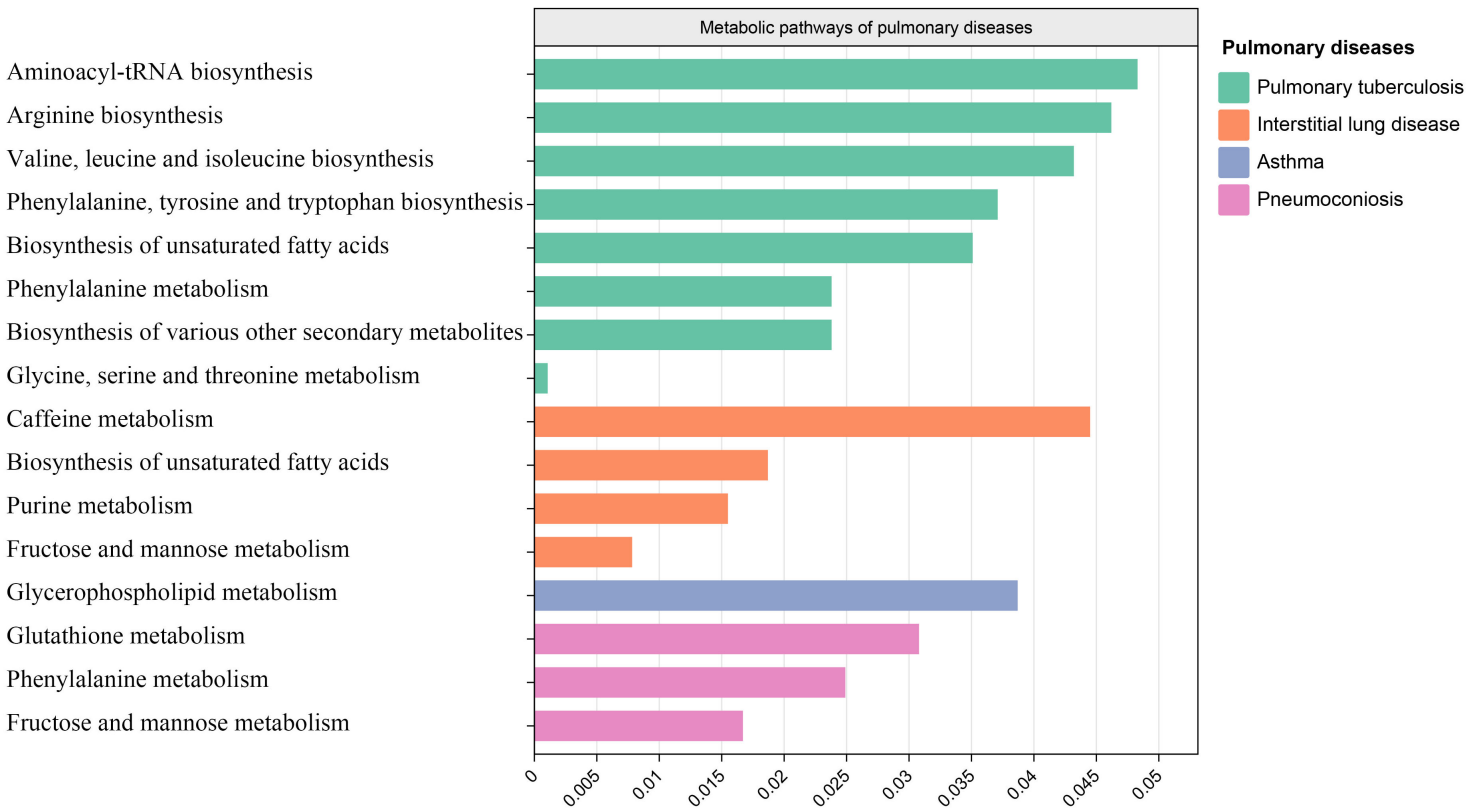
Metabolic pathways. Bar graph showing metabolic pathways enriched for metabolites causally associated with interstitial lung disease, tuberculosis, pneumoconiosis, asthma.

After analyzing the associated metabolites, we identified a total of 16 metabolic pathways that are integrated with SGLT2 and associated with pulmonary disease (**Figure 6**). There are four metabolic pathways associated with interstitial lung disease, with the most significant pathway being “Caffeine metabolism” (p=7.83×10^-3^). For pneumoconiosis, the most significant pathway among the three identified is “Glutathione metabolism” (p =1.67×10^-2^). In the case of pulmonary tuberculosis, among the eight metabolic pathways, the most significant one is “Aminoacyl-tRNA biosynthesis” (p = 1.07×10^-3^). Finally, for asthma, “Glycerophospholipid metabolism” (p=3.87×10^-2^) emerges as the only statistically significant. In addition, we noted a shared metabolic pathway, “Biosynthesis of unsaturated fatty acids”, between interstitial lung disease and pulmonary tuberculosis. Moreover, “Fructose and mannose metabolism” emerged as a common metabolic pathway between interstitial lung disease and pneumoconiosis, while “Phenylalanine metabolism” was identified as a shared pathway between pneumoconiosis and pulmonary tuberculosis (**Supplementary Table S4**).

### 3.7 Fine mapping and magma analysis

**Figure 7:**
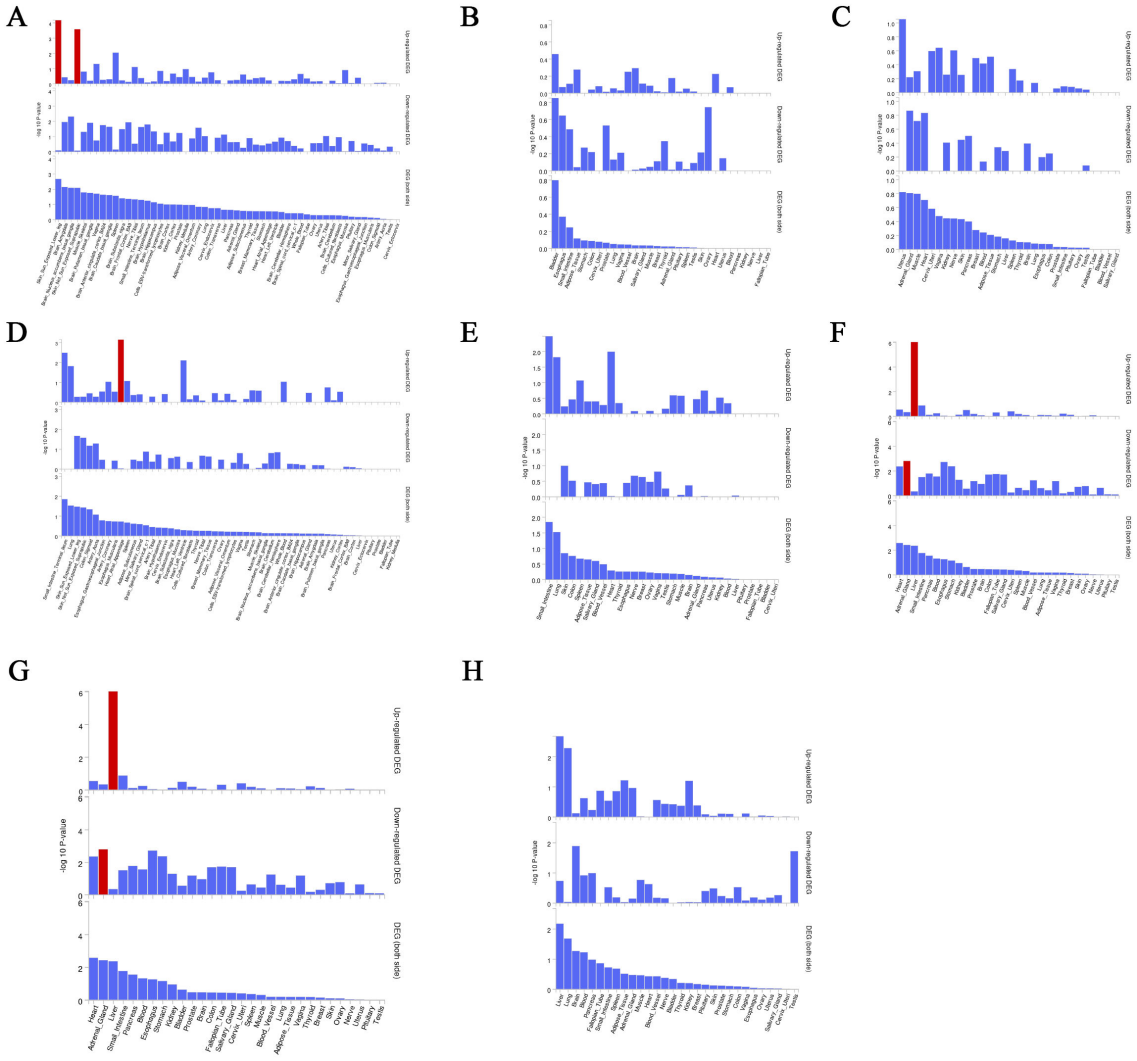
Magma analysis annotates the distribution of finely mapped DEG genes in tissues. Using the result of gene analysis (gene level p-value), (competitive) gene-set analysis is performed with default parameters with MAGMA v1.6. **(A)** Distribution of mapped genes in tissues of genus Phascolarctobacterium **(B)** Distribution of mapped genes in tissues of family Alcaligenaceae **(C)** Distribution of mapped genes in tissues of X-12719 **(D)** Distribution of mapped genes in tissues of family Enterobacteriaceae **(E)** Distribution of mapped genes in tissues of order Enterobacteriales **(F)** Distribution of mapped genes in tissues of Ratio of docosahexaenoic acid to total fatty acids **(G)** Distribution of mapped genes in tissues of Degree of unsaturation **(H)** Distribution of mapped genes in tissues of 4-androsten-3beta,17beta-diol disulfate 2.

We used the instrumental variable SNP for mediating metabolites and bacteriophages for fine mapping, leading to extensive identification of relevant target genes.16, 36, 63, and 1165 genes were mapped by FUMA in Interstitial lung disease, Pulmonary tuberculosis, Pneumoconiosis, and Asthma (**Supplementary Table S5**). Magma analysis then helped us to further identify the distribution of genes in tissues, and we found that in Pulmonary tuberculosis the differential genes mapped significantly in skin tissues; in Interstitial lung disease the differential genes mapped significantly in Heart tissues; and in Asthma the differential genes mapped significantly in Liver tissues. In Asthma, the differential gene mapped significantly in Liver tissue (**Figure 7**).

### 3.8 Multi-omics analysis to identify target genes

Proteins are important forms of function in the body. We used the cis-pQTL for analysis to identify positive proteins that were causally associated with outcomes (**Supplementary Table S6**). We found 71,66 and 71 causally associated proteins, but no overlap with the fine mapping results in Pulmonary tuberculosis, Pneumoconiosis and Interstitial lung disease; we found 125 causally associated proteins, with 6 overlapping proteins obtained from the fine mapping results in Asthma, and the results of the overlapping proteins are shown in **Table 2**. Proteins TCN2,TNFRSF1B and C10orf54 were positively associated with the development of asthma (B>0); proteins PDGFD,INHBC and ANXA7 were negatively associated with the development of asthma (B<0).

**Table 2:** Co-evidence of fine mapping and multi-omics.

Protein	Outcome	MR-Method	Beta	SE	Pval
ANXA7	**Asthma**	Maximum likelihood	-0.172	0.068	0.011
		Inverse variance weighted	-0.172	0.067	0.0097
TCN2		Inverse variance weighted	0.011	0.006	0.047
		MR Egger	0.0278	0.013	0.048
		Maximum likelihood	0.011	0.006	0.047
TNFRSF1B		Maximum likelihood	0.066	0.033	0.049
		Inverse variance weighted	0.066	0.033	0.048
C10orf54		Weighted median	0.059	0.019	0.002
		Inverse variance weighted	0.042	0.020	0.037
		Maximum likelihood	0.043	0.016	0.009
PDGFD		Maximum likelihood	-0.034	0.0170	0.042
		Weighted median	-0.045	0.022	0.037
		Inverse variance weighted	-0.035	0.0170	0.041
INHBC		Weighted median	-0.186	0.065	0.004
		Inverse variance weighted	-0.165	0.077	0.033
		Maximum likelihood	-0.172	0.056	0.002

### 3.9 Multiple effect evaluation of SGLT2 inhibitors across PheWAS analysis

**Figure 8:**
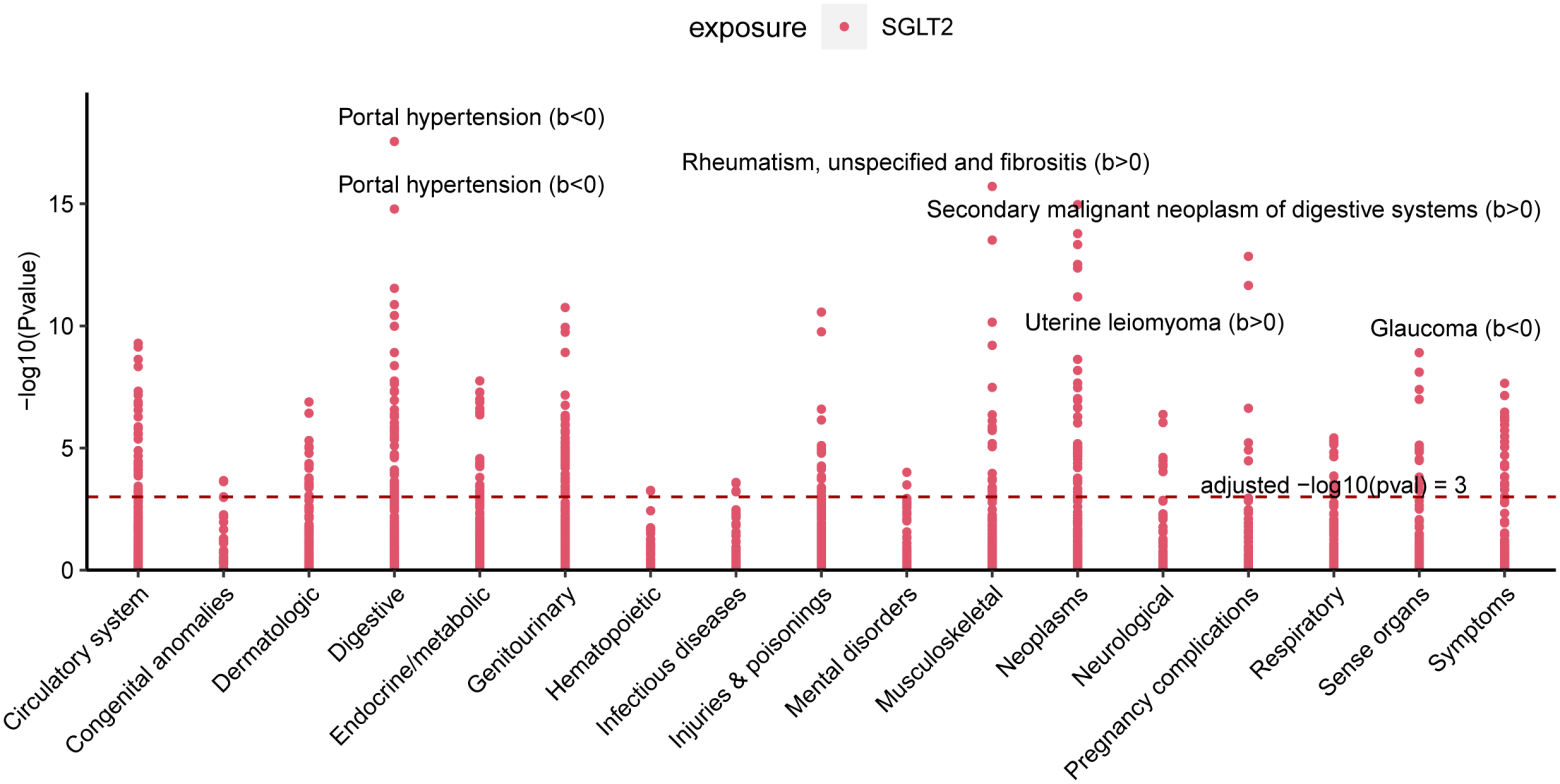
Multiple Effect Evaluation of SGLT2 Inhibitors Across PheWAS analysis. Manhattan plot showing phenotypic traits causally associated with instrumental variables for SGLT2. Horizontal coordinates indicate the categorization of phenotypes and vertical coordinates represent -log10 (Pvalue).

PheWAS analyses were performed using instrumental variables for SGLT2 inhibitors to determine their possible side effects and thus assess their effects more fully. After FDR correction, we found that SGLT2 inhibitors were associated with Portal hypertension (B<0, Pval=2.80E-18), Uterine leiomyoma (B>0, Pval=3.03E-13), Glaucoma (B<0, Pval=1.25E-09), Rheumatism, unspecified and fibrositis (B>0, Pval=1.94E-16) and Secondary malignant neoplasm of digestive systems (B>0, Pval=1.08E-15) had significant causal associations (**Figure 8**, **Supplementary Table S7**).

## 4 Discussion

**Figure 9:**
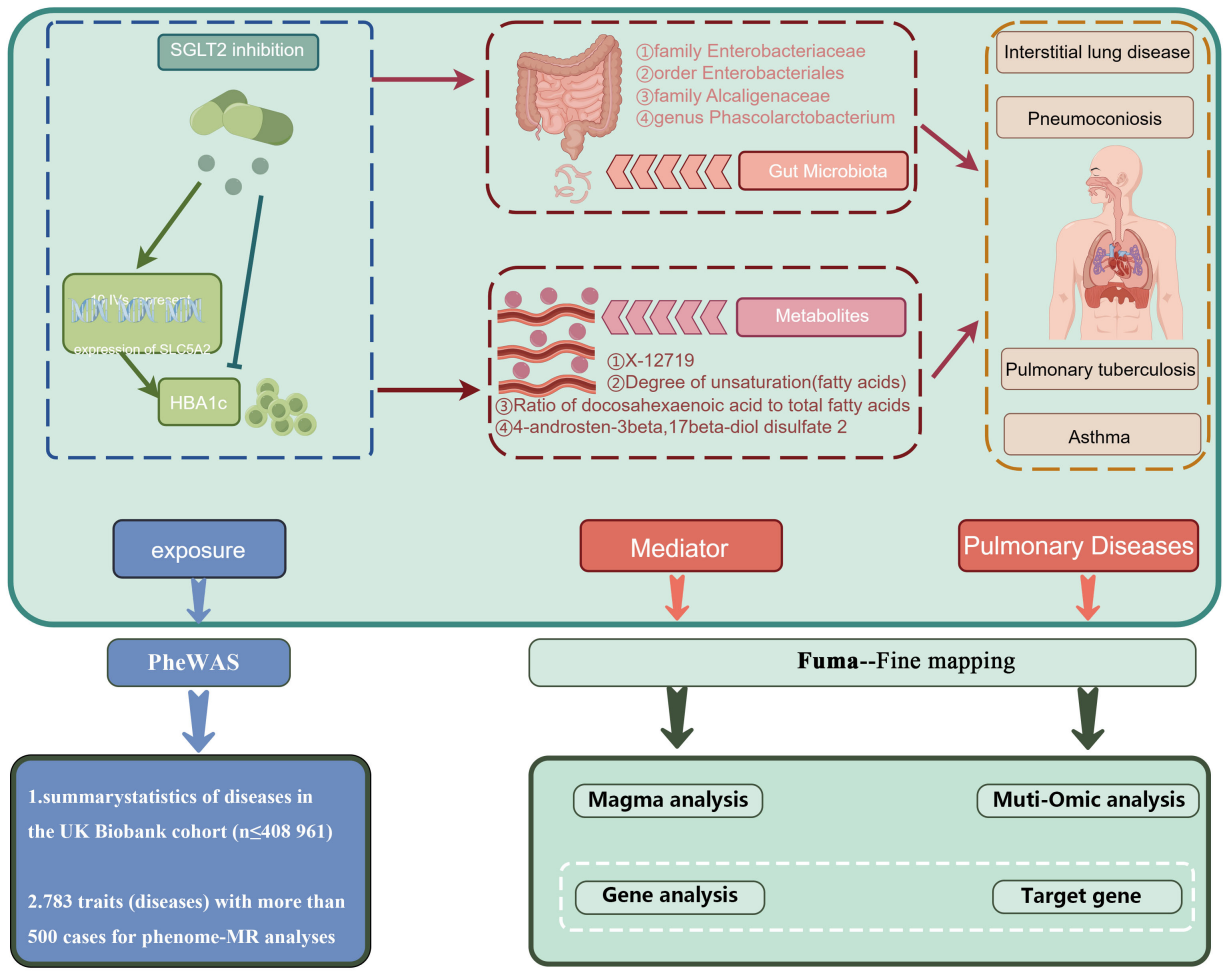
Mechanism role diagram. Mechanisms of SGLT2i action in lung disease inferred herein.

Over the past few decades, the rapid advancements in gut microbiota and metabolome studies have greatly enhanced our understanding of diseases. The discovery of the gut-lung axis and biomarkers such as metabolites has had a profound impact on the treatment and early diagnosis of diseases (Budden et al., 2017; Dang and Marsland, 2019). Our study is the first to comprehensively identify the lung-gut axis role of SGLT2 using a fine-mapping-based multi-omics research approach. We explored the potential mediating role of the gut microbiota and metabolites by examining the effects of SGLT2 inhibition on the gut microbiota and metabolites and their impact on lung-related diseases, and performed a multi-omics analysis to identify target genes that drive disease (**Figure 9**). In addition, our analyses identified 16 important metabolic pathways strongly associated with SGLT2 inhibition and the four lung diseases studied (interstitial lung disease, pneumoconiosis, tuberculosis, and asthma), and comprehensively assessed the possible side effects of SGLT2 inhibitors.

### 4.1 The relationship between SGLT2 inhibition and ten pulmonary diseases

Some previous clinical trials, cohort studies, and retrospective analyses have investigated the role of SGLT2 inhibition in pulmonary diseases. They found that SGLT2 inhibitors may reduce the risk of asthma, pneumonia, exacerbations in patients with chronic obstructive pulmonary disease, improve pulmonary arterial hypertension in patients with interstitial lung disease, and serve as a therapeutic target for early-stage lung adenocarcinoma (Scafoglio et al., 2018; Qiu et al., 2021; Pradhan et al., 2022; Wu et al., 2022). However, there is still controversy surrounding their results. Moreover, there remains a paucity of research on the correlation between SGLT2 inhibitors and pneumoconiosis, pulmonary tuberculosis. Our MR study results affirm the risk reduction in asthma and interstitial lung disease, supporting the aforementioned associations. However, no favorable outcomes were discerned for COPD, pneumonia, and lung cancer. The precise mechanisms underlying the protective effects of SGLT2 inhibitors against respiratory diseases have not been fully elucidated thus far. In a murine model of asthma, it was observed that SGLT2 inhibitors could reduce airway hyperreactivity, ameliorate airway inflammation and remodeling. In the microscopic structure of the lungs, they alleviated thickening of the bronchiolar epithelium, hyperplasia of goblet cells, fibrosis, and hypertrophy of smooth muscles (Hussein et al., 2023). Additionally, SGLT2 inhibitors were found to possess anti-pulmonary fibrosis effects (Park et al., 2019). Finally, our study results also revealed a decreased risk of pneumoconiosis and pulmonary tuberculosis with SGLT2 inhibition. However, further validation is required in future research.

### 4.2 The relationship between SGLT2 inhibition and gut microbiota, as well as circulating metabolites

In animal models, the significant SGLT2 inhibitors, dapagliflozin, and empagliflozin, have been observed to induce changes in the gut microbiota, impacting the progression of various diseases. Empagliflozin, for instance, reduces lipopolysaccharide-producing bacteria like Oscillibacter while increasing short-chain fatty acid-producing bacteria such as Bacteroid and Odoribacter, thereby contributing to the amelioration of diabetic nephropathy (Deng et al., 2022). Similarly, dapagliflozin is associated with improvements in arterial stiffness, vascular smooth muscle function, and alterations in the composition of gut microbiota, including Actinobacteria, Bacteroidetes, Firmicutes, Verrucomicrobia and Proteobacteria (Lee et al., 2018). However, in human studies, no significant changes in the gut microbiota composition were observed in fecal samples after 12 weeks of treatment with dapagliflozin (van Bommel et al., 2020). These studies might be affected by remaining confounding factors, and the precise alterations in the gut microbiota induced by SGLT2 inhibitors remain unclear. Hence, our MR study delved into the relationship between SGLT2 inhibition and the gut microbiota, uncovering modifications in 152 gut microbiota. Further research is needed to elucidate the connections between these microbiota changes and various diseases.

The impact of SGLT2 inhibitors on metabolites has been observed in early studies. The SGLT2 inhibitors were initially discovered to induce changes in lipid metabolites. In diabetic mouse models, the use of canagliflozin was associated with a reduction in circulating cholesterol (Osataphan et al., 2019), and treating high-fat-fed mice with dapagliflozin inhibited lipid accumulation (Sato et al., 2022). In a meta-analysis study, SGLT2 inhibitors have been shown to alter levels of total cholesterol, low-density lipoprotein cholesterol, non-high-density lipoprotein cholesterol, high-density lipoprotein cholesterol, and triglycerides (Sánchez-García et al., 2020). The impact of SGLT2 inhibition extends to other blood metabolites, as evidenced by a study measuring plasma metabolite changes in patients treated with empagliflozin, revealing significant alterations in the tricarboxylic acid cycle, unsaturated fatty acids, butyric acid, propionic acid, alanine, aspartic acid, and glutamic acid (Liu et al., 2021). However, there is ongoing debate about how metabolites are altered. Therefore, in our study, we utilized SGLT2 genetic variants as instrumental variables to explore changes in metabolites. To identify more metabolite changes, we employed two metabolite GWAS datasets—one predominantly containing lipid metabolites and the other encompassing various blood metabolites. Our study results revealed changes in 173 out of 249 metabolites and 220 out of 486 metabolites in the two respective datasets. The connections between metabolites and multiple diseases still require further exploration.

### 4.3 The mediating role of gut microbiota and metabolites in the association between SGLT2 inhibition and interstitial lung disease, pneumoconiosis, pulmonary tuberculosis, and asthma

The aforementioned studies have already revealed that SGLT2 inhibitors induce changes in gut microbiota and metabolites. The investigation into the role of gut microbiota and metabolites in pulmonary diseases is currently progressing vigorously.

Our research results indicate that the family Enterobacteriaceae and the order Enterobacteriales play a mediating role in interstitial lung disease. These two bacteria have not been identified in current research on interstitial lung disease. In a study predominantly involving females with interstitial lung disease, the dysregulation of gut microbiota was identified as a significant factor exacerbating the disease (Chioma et al., 2023). However, the specific bacteria responsible for the dysregulation have not been elucidated. Currently, there is limited data on the gut microbiota of patients with interstitial lung disease. In our study, in addition to these two clearly identified intermediate bacterial groups, there are also some significant bacteria. However, these bacteria still require further investigation in future studies. Additionally, in the study of metabolites and interstitial lung disease, while no mediating metabolites were identified, there are several significant metabolites, such as X-11422 (xanthine), X-14208 (phenylalanylserine), and Gamma-glutamyltyrosine. In a study involving blood samples related to interstitial lung disease in systemic sclerosis, xanthine was found to distinguish the severity of the condition (Meier et al., 2020). Metabolomic research on cellular metabolism in lung fibrosis revealed changes in various amino acids such as glutamine, glutamate, glycine, and lipid metabolites (Roque and Romero, 2021). While our study partially corroborates these findings, we also identified additional metabolites that require further investigation. Furthermore, our research uncovered numerous lipid metabolites associated with interstitial pneumonia; however, they did not maintain significance after multiple corrections and are therefore not discussed in our analysis. We hope that future studies will provide better insights to validate these observations.

Our research findings suggest that the family Alcaligenaceae and X-12719 play a mediating role in pneumoconiosis. In a Mendelian randomization study investigating the relationship between gut microbiota and pneumoconiosis, utilizing a database from Finland, we also identified a significant association between the Alcaligenaceae family and pneumoconiosis (Shi et al., 2023). This bacterium has not been previously discovered in pneumoconiosis research. Another significant bacterium in the results was Ruminococcaceae, but it did not act as a mediator. A similar finding was observed in a pneumoconiosis rat study, where they observed changes in bacteria such as Ruminococcaceae NK4A214 group, Ruminiclostridium 5, Allobaculum, among others (Guo et al., 2023). The involvement of other bacteria identified in the results still requires further confirmation. Regarding metabolites, our result indicates an unknown metabolite in an intermediate position, which requires further clarification in the future. In our study, we also identified some significant metabolites that did not act as intermediates, such as Erythrose, N-acetylalanine, Gamma-glutamylleucine, etc. Changes in amino acids and peptides have been observed in previous studies on pneumoconiosis patients (Li et al., 2022b). Notably, N-acetylalanine and Gamma-glutamylleucine belong to the category of amino acids and peptides. Therefore, the metabolites we discovered, combined with findings from previous research, can contribute to future studies on pneumoconiosis.

Our research findings suggest that the genus Phascolarctobacterium is the only one playing a mediating role in pneumoconiosis. In a study focusing on the gut microbiota of tuberculosis patients, significant enrichment of Phascolarctobacterium, Eubacterium, Faecalibacterium, and Roseburia was observed (Maji et al., 2018). This finding provides strong validation for our research results. Additionally, in another study involving 16S rRNA gene sequencing of fecal samples from tuberculosis patients, Eubacterium was identified as a diagnostic biomarker for tuberculosis (Ye et al., 2022). Although Eubacterium lost statistical significance in our study results after multiple corrections, considering that multiple corrections are a stringent correction method, it may lead to the loss of some potential positive findings. Therefore, in the future, integrating our microbiota results may provide a basis for more detailed studies on the treatment of pulmonary tuberculosis patients. Regarding metabolites, all the metabolites in our study lost statistical significance after multiple corrections. In metabolomic studies of pulmonary tuberculosis patients, a decrease in levels of cholesterol and other lipid metabolites (Wang et al., 2022), as well as changes in some amino acid levels (Szeszko et al., 2007), have been observed. Similar changes in these metabolites were also noted in our results. Therefore, further research is needed to explore metabolites in studies involving pulmonary tuberculosis patients.

Our research indicates that the degree of unsaturation (fatty acids), ratio of docosahexaenoic acid to total fatty acids, and 4-androsten-3beta,17beta-diol disulfate 2* serve as mediating factor in asthma. Our results did not reveal mediating gut microbiota, but some significant bacteria were identified, such as Barnesiella, Lachnospiraceae, Holdemanella, Bacteroidaceae, Bacteroides, etc. The alterations of these bacteria have been reported in asthma patients or mouse models of asthma (Zimmermann et al., 2019; Gu et al., 2023; Liu et al., 2023a). Regarding mediating metabolites, it has been demonstrated in mouse models that controlling the metabolism of long-chain unsaturated fatty acids can alter airway inflammation, thereby influencing allergic asthma (Hou et al., 2022). In a study on infant growth and development, the addition of polyunsaturated fatty acids, docosahexaenoic acid, and arachidonic acid to formula can reduce the incidence of asthma (Miles et al., 2021). There are also numerous studies on fatty acids and asthma, with fatty acids playing a crucial role in the development and resolution of inflammation pathways associated with asthma (Wendell et al., 2014; Garcia-Larsen, 2021). Another mediating metabolite, 4-androsten-3beta,17beta-diol disulfate 2*, belongs to lipid metabolites. Current research suggests its potential association with stroke (Zhang et al., 2023), but there is currently no research on its correlation with asthma. Lipid metabolites identified in our research can validate previous studies on metabolites related to asthma patients and provide some yet undiscovered metabolites. However, further validation of their association with asthma is still needed in future research.

### 4.4 The metabolic pathways in the association between SGLT2 inhibition and interstitial lung disease, pneumoconiosis, pulmonary tuberculosis, and asthma

Our MR analysis identified metabolic pathways that exhibit a causal relationship with the development of interstitial lung disease, pneumoconiosis, pulmonary tuberculosis, and asthma. In interstitial lung disease, four metabolic pathways were discovered, including the caffeine metabolism, biosynthesis of unsaturated fatty acids, purine metabolism and fructose and mannose metabolism. These four metabolic pathways have not been previously reported in studies on interstitial lung disease. In our results, the primary metabolites associated with caffeine metabolism and purine metabolism include xanthine, as discussed earlier. The “Biosynthesis of unsaturated fatty acids” pathway is commonly present in both interstitial lung disease and pulmonary tuberculosis, with the main metabolite involved being Eicosapentaenoate. While Eicosapentaenoate has not been reported in these two diseases, it belongs to the category of unsaturated fatty acids, which have been previously studied in the context of both interstitial lung disease and pulmonary tuberculosis (Yi et al., 2019; Kim et al., 2021).

The metabolic pathways identified in pneumoconiosis include glutathione metabolism, phenylalanine metabolism, and fructose and mannose metabolism. The metabolic pathway of “Fructose and mannose metabolism” is shared between interstitial lung disease and pneumoconiosis, with Mannitol being a key metabolite involved. The glutathione metabolism and phenylalanine metabolism involved metabolites are 5-oxoproline and 3-(3-hydroxyphenyl)propionate. The three metabolites mentioned, have not been reported in the existing literature. Both 5-oxoproline and 3-(3-hydroxyphenyl)propionate belong to amino acids, and alterations in amino acids have been reported in studies involving pneumoconiosis patients (Li et al., 2022b; Kan et al., 2023).

The metabolic pathways found to be associated with pulmonary tuberculosis encompass Aminoacyl-tRNA biosynthesis, Arginine biosynthesis, Valine, leucine and isoleucine biosynthesis, Phenylalanine, tyrosine and tryptophan biosynthesis, Biosynthesis of unsaturated fatty acids, Phenylalanine metabolism, Biosynthesis of various other secondary metabolites, and Glycine, serine and threonine metabolism. The key metabolites implicated in these pathways include Phenylalanine, Threonine, Citrulline, and Eicosapentaenoate. As previously discussed, Eicosapentaenoate has been covered earlier, and the remaining three metabolites all belong to amino acids, with reported associations in studies related to tuberculosis (Lima et al., 2013; Das et al., 2015; Li et al., 2022a).

The only identified metabolic pathway in asthma is Glycerophospholipid metabolism. In previous plasma lipidomics studies on asthma patients, Glycerophospholipid metabolism has been revealed to play a crucial role in asthma. Our research findings strongly validate the aforementioned discoveries. The main metabolite involved in this pathway is 1-myristoylglycerophosphocholine, which currently lacks relevant reports.

Additionally, disparities exist between the metabolites predominantly implicated in pathway analysis and those highlighted in our significant results. This suggests the presence of potentially overlooked metabolic pathways. Further investigation is essential to gain a more comprehensive understanding of the interconnections among metabolites, metabolic pathways, and pulmonary diseases.

### 4.5 Fine mapping and multi-omics analysis

Mediation analysis based on metabolites and gut flora does not allow for more in-depth determination of the mechanisms by which they affect disease. That is why it is said that with Fuma it is possible to identify very efficiently the genes that are annotated and mapped for further evaluation with the disease (Liu et al., 2023b). This study accordingly identified multiple mapping sets for four lung diseases. Six effector genes that overlap in asthma were further identified by proteomic MR analysis.

### 4.6 Strengths and limitations

Our study is the first to comprehensively identify the lung-gut axis role of SGLT2 using a fine-mapping-based multi-omics research approach. However, it is important to recognize some inherent limitations of our study. First, the study participants were predominantly of European descent, and thus the generalizability of our findings to other populations needs to be assessed. Second, metabolites from the two data sources may overlap, which may bias the results. Finally, although MR has helped to determine the effects of SGLT2 inhibition on the gut microbiota, metabolites, and genes, which in turn affect associated lung diseases, prospective or randomized controlled studies are needed to delve deeper into the underlying mechanisms.

## 5 Conclusion

This comprehensive study strongly supports a multi-omics association between SGLT2 inhibition and reduced risk of interstitial lung disease, tuberculosis, pneumoconiosis, and asthma. Four identified gut microbiota, four metabolites, 16 metabolic pathways, and six target genes appear to play a potential role in this association. The results of the comprehensive phenome-wide association analysis also identified the full effect of SGLT2 inhibitors.

## Data Availability

The datasets presented in this study can be found in online repositories. The names of the repository/repositories and accession number(s) can be found in the article/**Supplementary Material**.

## References

[B1] Budden K. F. Gellatly S. L. Wood D. L. Cooper M. A. Morrison M. Hugenholtz P.. (2017). Emerging pathogenic links between microbiota and the gut-lung axis. Nat. Rev. Microbiol. 15, 55–63. doi: 10.1038/nrmicro.2016.142 27694885

[B2] Bycroft C. Freeman C. Petkova D. Band G. Elliott L. T. Sharp K.. (2018). The UK Biobank resource with deep phenotyping and genomic data. Nature 562, 203–209. doi: 10.1038/s41586-018-0579-z 30305743 PMC6786975

[B3] Chioma O. S. Mallott E. Shah-Gandhi B. Wiggins Z. Langford M. Lancaster A. W.. (2023). Low gut microbial diversity augments estrogen-driven pulmonary fibrosis in female-predominant interstitial lung disease. Cells 12 (5), 766. doi: 10.3390/cells12050766 36899902 PMC10000459

[B4] Cowie M. R. Fisher M. (2020). SGLT2 inhibitors: mechanisms of cardiovascular benefit beyond glycaemic control. Nat. Rev. Cardiol. 17, 761–772. doi: 10.1038/s41569-020-0406-8 32665641

[B5] Dai H. Zheng L. Zhu Z. Geng X. Hou T. Wang Q.. (2023). Evaluation of the effect of sodium-glucose cotransporter 2 inhibition on fracture risk: evidence from mendelian randomization and genetic association study. J. Bone Miner Res. 38 (11), 1645-1653. doi: 10.1002/jbmr.4880 37436694

[B6] Dang A. T. Marsland B. J. (2019). Microbes, metabolites, and the gut-lung axis. Mucosal Immunol. 12, 843–850. doi: 10.1038/s41385-019-0160-6 30976087

[B7] Das M. K. Bishwal S. C. Das A. Dabral D. Badireddy V. K. Pandit B.. (2015). Deregulated tyrosine-phenylalanine metabolism in pulmonary tuberculosis patients. J. Proteome Res. 14, 1947–1956. doi: 10.1021/acs.jproteome.5b00016 25693719

[B8] Deng L. Yang Y. Xu G. (2022). Empagliflozin ameliorates type 2 diabetes mellitus-related diabetic nephropathy via altering the gut microbiota. Biochim. Biophys. Acta Mol. Cell Biol. Lipids 1867, 159234. doi: 10.1016/j.bbalip.2022.159234 36185030

[B9] Ferkingstad E. Sulem P. Atlason B. A. Sveinbjornsson G. Magnusson M. I. Styrmisdottir E. L.. (2021). Large-scale integration of the plasma proteome with genetics and disease. Nat. Genet. 53, 1712–1721. doi: 10.1038/s41588-021-00978-w 34857953

[B10] Garcia-Larsen V. (2021). Omega-3 polyunsaturated fatty acids and FADS genotype: is personalised prevention of asthma on the horizon? Eur. Respir. J. 58 (3), 2101386. doi: 10.1183/13993003.01386-2021 34475115

[B11] Georgakis M. K. Gill D. (2021). Mendelian randomization studies in stroke: exploration of risk factors and drug targets with human genetic data. Stroke 52, 2992–3003. doi: 10.1161/strokeaha.120.032617 34399585

[B12] GTEx Consortium. (2020). The GTEx Consortium atlas of genetic regulatory effects across human tissues. Science 369, 1318–1330. doi: 10.1126/science.aaz1776 32913098 PMC7737656

[B13] Gu B. H. Choi J. P. Park T. Kim A. S. Jung H. Y. Choi D. Y.. (2023). Adult asthma with symptomatic eosinophilic inflammation is accompanied by alteration in gut microbiome. Allergy 78, 1909–1921. doi: 10.1111/all.15691 36847620

[B14] Guo J. Zhang B. Xiong Y. Kang T. Han Y. Xu Y.. (2023). The temporal characteristics of the disruption of gut microbiota, serum metabolome, and cytokines by silica exposure in wistar rats. Ecotoxicol Environ. Saf. 252, 114580. doi: 10.1016/j.ecoenv.2023.114580 36706523

[B15] Herat L. Y. Ward N. C. Magno A. L. Rakoczy E. P. Kiuchi M. G. Schlaich M. P.. (2020). Sodium glucose co-transporter 2 inhibition reduces succinate levels in diabetic mice. World J. Gastroenterol. 26, 3225–3235. doi: 10.3748/wjg.v26.i23.3225 32684737 PMC7336319

[B16] Hou Y. Wei D. Zhang Z. Guo H. Li S. Zhang J.. (2022). FABP5 controls macrophage alternative activation and allergic asthma by selectively programming long-chain unsaturated fatty acid metabolism. Cell Rep. 41, 111668. doi: 10.1016/j.celrep.2022.111668 36384126

[B17] Hussein N. A. Abdel Gawad H. S. Maklad H. M. El-Fakharany E. M. Aly R. G. Samy D. M. (2023). Empagliflozin inhibits autophagy and mitigates airway inflammation and remodelling in mice with ovalbumin-induced allergic asthma. Eur. J. Pharmacol. 950, 175701. doi: 10.1016/j.ejphar.2023.175701 37044313

[B18] Johnson C. H. Ivanisevic J. Siuzdak G. (2016). Metabolomics: beyond biomarkers and towards mechanisms. Nat. Rev. Mol. Cell Biol. 17, 451–459. doi: 10.1038/nrm.2016.25 26979502 PMC5729912

[B19] Kan L. H. Xu X. Chen Y. M. Wang X. M. Li J. L. Shen F. H. (2023). Correlation between intestinal and respiratory flora and their metabolites in a rat pneumoconiosis model. Zhonghua Lao Dong Wei Sheng Zhi Ye Bing Za Zhi 41, 21–30. doi: 10.3760/cma.j.cn121094-20211011-00495 36725290

[B20] Kappel B. A. Lehrke M. Schütt K. Artati A. Adamski J. Lebherz C.. (2017). Effect of empagliflozin on the metabolic signature of patients with type 2 diabetes mellitus and cardiovascular disease. Circulation 136, 969–972. doi: 10.1161/circulationaha.117.029166 28874423

[B21] Kim J. S. Steffen B. T. Podolanczuk A. J. Kawut S. M. Noth I. Raghu G.. (2021). Associations of ω-3 fatty acids with interstitial lung disease and lung imaging abnormalities among adults. Am. J. Epidemiol. 190, 95–108. doi: 10.1093/aje/kwaa168 32803215 PMC7784523

[B22] Kintu C. Soremekun O. Kamiza A. B. Kalungi A. Mayanja R. Kalyesubula R.. (2023). The causal effects of lipid traits on kidney function in Africans: bidirectional and multivariable Mendelian-randomization study. EBioMedicine 90, 104537. doi: 10.1016/j.ebiom.2023.104537 37001235 PMC10070509

[B23] Kurilshikov A. Medina-Gomez C. Bacigalupe R. Radjabzadeh D. Wang J. Demirkan A.. (2021). Large-scale association analyses identify host factors influencing human gut microbiome composition. Nat. Genet. 53, 156–165. doi: 10.1038/s41588-020-00763-1 33462485 PMC8515199

[B24] Kurki M. I. Karjalainen J. Palta P. Sipilä T. P. Kristiansson K. Donner K. M.. (2023). FinnGen provides genetic insights from a well-phenotyped isolated population. Nature 613, 508–518. doi: 10.1038/s41586-022-05473-8 36653562 PMC9849126

[B25] Kwok M. K. Kawachi I. Rehkopf D. Schooling C. M. (2020). The role of cortisol in ischemic heart disease, ischemic stroke, type 2 diabetes, and cardiovascular disease risk factors: a bi-directional Mendelian randomization study. BMC Med. 18, 363. doi: 10.1186/s12916-020-01831-3 33243239 PMC7694946

[B26] Lee D. M. Battson M. L. Jarrell D. K. Hou S. Ecton K. E. Weir T. L.. (2018). SGLT2 inhibition via dapagliflozin improves generalized vascular dysfunction and alters the gut microbiota in type 2 diabetic mice. Cardiovasc. Diabetol. 17, 62. doi: 10.1186/s12933-018-0708-x 29703207 PMC5921754

[B27] Li J. Yu Y. Sun Y. Yu B. Tan X. Wang B.. (2023). SGLT2 inhibition, circulating metabolites, and atrial fibrillation: a Mendelian randomization study. Cardiovasc. Diabetol. 22, 278. doi: 10.1186/s12933-023-02019-8 37848934 PMC10583416

[B28] Li Q. Peng Z. Fu X. Wang H. Zhao Z. Pang Y.. (2022a). Rv3737 is required for Mycobacterium tuberculosis growth in *vitro* and in *vivo* and correlates with bacterial load and disease severity in human tuberculosis. BMC Infect. Dis. 22, 256. doi: 10.1186/s12879-021-06967-y 35287590 PMC8919692

[B29] Li Y. Xiao K. Xiao S. Wang M. Pei S. Liu H.. (2022b). Difference in intestinal flora and characteristics of plasma metabonomics in pneumoconiosis patients. Metabolites 12 (10), 917. doi: 10.3390/metabo12100917 36295819 PMC9609413

[B30] Lima I. Oliveira R. C. Atta A. Marchi S. Barbosa L. Reis E.. (2013). Antibodies to citrullinated peptides in tuberculosis. Clin. Rheumatol 32, 685–687. doi: 10.1007/s10067-013-2173-y 23344687

[B31] Liu F. Duan W. Guan T. Zhou Q. Yan W. Geng Y. (2023a). Water extract of Pingchuan formula ameliorated murine asthma through modulating metabolites and gut microbiota. J. Pharm. BioMed. Anal. 236, 115728. doi: 10.1016/j.jpba.2023.115728 37793314

[B32] Liu X. Miao Y. Liu C. Lu W. Feng Q. Zhang Q. (2023b). Identification of multiple novel susceptibility genes associated with autoimmune thyroid disease. Front. Immunol. 14. doi: 10.3389/fimmu.2023.1161311 PMC10183592 37197658

[B33] Liu H. Sridhar V. S. Montemayor D. Lovblom L. E. Lytvyn Y. Ye H.. (2021). Changes in plasma and urine metabolites associated with empagliflozin in patients with type 1 diabetes. Diabetes Obes. Metab. 23, 2466–2475. doi: 10.1111/dom.14489 34251085

[B34] Maji A. Misra R. Dhakan D. B. Gupta V. Mahato N. K. Saxena R.. (2018). Gut microbiome contributes to impairment of immunity in pulmonary tuberculosis patients by alteration of butyrate and propionate producers. Environ. Microbiol. 20, 402–419. doi: 10.1111/1462-2920.14015 29322681

[B35] Meier C. Freiburghaus K. Bovet C. Schniering J. Allanore Y. Distler O.. (2020). Serum metabolites as biomarkers in systemic sclerosis-associated interstitial lung disease. Sci. Rep. 10, 21912. doi: 10.1038/s41598-020-78951-6 33318574 PMC7736572

[B36] Miles E. A. Childs C. E. Calder P. C. (2021). Long-chain polyunsaturated fatty acids (LCPUFAs) and the developing immune system: A narrative review. Nutrients 13 (1), 247. doi: 10.3390/nu13010247 33467123 PMC7830895

[B37] Mohus R. M. Flatby H. Liyanarachi K. V. DeWan A. T. Solligård E. Damås J. K.. (2022). Iron status and the risk of sepsis and severe COVID-19: a two-sample Mendelian randomization study. Sci. Rep. 12, 16157. doi: 10.1038/s41598-022-20679-6 36171422 PMC9516524

[B38] Osataphan S. Macchi C. Singhal G. Chimene-Weiss J. Sales V. Kozuka C.. (2019). SGLT2 inhibition reprograms systemic metabolism via FGF21-dependent and -independent mechanisms. JCI Insight 4 (5), e123130. doi: 10.1172/jci.insight.123130 30843877 PMC6483601

[B39] Park S. L. Fesinmeyer M. D. Timofeeva M. Caberto C. P. Kocarnik J. M. Han Y.. (2014). Pleiotropic associations of risk variants identified for other cancers with lung cancer risk: the PAGE and TRICL consortia. J. Natl. Cancer Inst 106, dju061. doi: 10.1093/jnci/dju061 24681604 PMC3982896

[B40] Park H. J. Han H. Oh E. Y. Kim S. R. Park K. H. Lee J. H.. (2019). Empagliflozin and dulaglutide are effective against obesity-induced airway hyperresponsiveness and fibrosis in A murine model. Sci. Rep. 9, 15601. doi: 10.1038/s41598-019-51648-1 31666643 PMC6821734

[B41] Pradhan R. Lu S. Yin H. Yu O. H. Y. Ernst P. Suissa S.. (2022). Novel antihyperglycaemic drugs and prevention of chronic obstructive pulmonary disease exacerbations among patients with type 2 diabetes: population based cohort study. Bmj 379, e071380. doi: 10.1136/bmj-2022-071380 36318979 PMC9623550

[B42] Qiu S. Cai Y. Yao H. Lin C. Xie Y. Tang S.. (2023). Small molecule metabolites: discovery of biomarkers and therapeutic targets. Signal Transduct Target Ther. 8, 132. doi: 10.1038/s41392-023-01399-3 36941259 PMC10026263

[B43] Qiu M. Ding L. L. Zhan Z. L. Liu S. Y. (2021). Use of SGLT2 inhibitors and occurrence of noninfectious respiratory disorders: a meta-analysis of large randomized trials of SGLT2 inhibitors. Endocrine 73, 31–36. doi: 10.1007/s12020-021-02644-x 33559806

[B44] Ritchie S. C. Surendran P. Karthikeyan S. Lambert S. A. Bolton T. Pennells L.. (2023). Quality control and removal of technical variation of NMR metabolic biomarker data in ~120,000 UK Biobank participants. Sci. Data 10, 64. doi: 10.1038/s41597-023-01949-y 36720882 PMC9887579

[B45] Roque W. Romero F. (2021). Cellular metabolomics of pulmonary fibrosis, from amino acids to lipids. Am. J. Physiol. Cell Physiol. 320, C689–c695. doi: 10.1152/ajpcell.00586.2020 33471621 PMC8163573

[B46] Sakaue S. Kanai M. Tanigawa Y. Karjalainen J. Kurki M. Koshiba S.. (2021). A cross-population atlas of genetic associations for 220 human phenotypes. Nat. Genet. 53, 1415–1424. doi: 10.1038/s41588-021-00931-x 34594039 PMC12208603

[B47] Sánchez-García A. Simental-Mendía M. Millán-Alanís J. M. Simental-Mendía L. E. (2020). Effect of sodium-glucose co-transporter 2 inhibitors on lipid profile: A systematic review and meta-analysis of 48 randomized controlled trials. Pharmacol. Res. 160, 105068. doi: 10.1016/j.phrs.2020.105068 32652200

[B48] Sato D. Nakamura T. Amarume J. Yano M. Umehara Y. Nishina A.. (2022). Effects of dapagliflozin on adipose and liver fatty acid composition and mRNA expression involved in lipid metabolism in high-fat-fed rats. Endocr. Metab. Immune Disord. Drug Targets 22, 944–953. doi: 10.2174/1871530322666220307153618 35255800

[B49] Scafoglio C. R. Villegas B. Abdelhady G. Bailey S. T. Liu J. Shirali A. S.. (2018). Sodium-glucose transporter 2 is a diagnostic and therapeutic target for early-stage lung adenocarcinoma. Sci. Transl. Med. 10 (467), eaat5933. doi: 10.1126/scitranslmed.aat5933 30429355 PMC6428683

[B50] Sedgwick P. (2014). Multiple hypothesis testing and Bonferroni’s correction. Bmj 349, g6284. doi: 10.1136/bmj.g6284 25331533

[B51] Shi H. Zhao T. Geng R. Sun L. Fan H. (2023). The associations between gut microbiota and chronic respiratory diseases: a Mendelian randomization study. Front. Microbiol. 14. doi: 10.3389/fmicb.2023.1200937 PMC10272395 37333634

[B52] Shin S. Y. Fauman E. B. Petersen A. K. Krumsiek J. Santos R. Huang J.. (2014). An atlas of genetic influences on human blood metabolites. Nat. Genet. 46, 543–550. doi: 10.1038/ng.2982 24816252 PMC4064254

[B53] Storey J. D. Tibshirani R. (2003). Statistical significance for genomewide studies. Proc. Natl. Acad. Sci. U.S.A. 100, 9440–9445. doi: 10.1073/pnas.1530509100 12883005 PMC170937

[B54] Szekeres Z. Toth K. Szabados E. (2021). The effects of SGLT2 inhibitors on lipid metabolism. Metabolites 11 (2), 87. doi: 10.3390/metabo11020087 33535652 PMC7912792

[B55] Szeszko J. S. Healy B. Stevens H. Balabanova Y. Drobniewski F. Todd J. A.. (2007). Resequencing and association analysis of the SP110 gene in adult pulmonary tuberculosis. Hum. Genet. 121, 155–160. doi: 10.1007/s00439-006-0293-z 17149599

[B56] van Bommel E. J. M. Herrema H. Davids M. Kramer M. H. H. Nieuwdorp M. van Raalte D. H. (2020). Effects of 12-week treatment with dapagliflozin and gliclazide on faecal microbiome: Results of a double-blind randomized trial in patients with type 2 diabetes. Diabetes Metab. 46, 164–168. doi: 10.1016/j.diabet.2019.11.005 31816432

[B57] Verbanck M. Chen C. Y. Neale B. Do R. (2018). Detection of widespread horizontal pleiotropy in causal relationships inferred from Mendelian randomization between complex traits and diseases. Nat. Genet. 50, 693–698. doi: 10.1038/s41588-018-0099-7 29686387 PMC6083837

[B58] Võsa U. Claringbould A. Westra H. J. Bonder M. J. Deelen P. Zeng B.. (2021). Large-scale cis- and trans-eQTL analyses identify thousands of genetic loci and polygenic scores that regulate blood gene expression. Nat. Genet. 53, 1300–1310. doi: 10.1038/s41588-021-00913-z 34475573 PMC8432599

[B59] Wang S. Yang L. Hu H. Lv L. Ji Z. Zhao Y.. (2022). Characteristic gut microbiota and metabolic changes in patients with pulmonary tuberculosis. Microb. Biotechnol. 15, 262–275. doi: 10.1111/1751-7915.13761 33599402 PMC8719804

[B60] Watanabe K. Taskesen E. van Bochoven A. Posthuma D. (2017). Functional mapping and annotation of genetic associations with FUMA. Nat. Commun. 8, 1826. doi: 10.1038/s41467-017-01261-5 29184056 PMC5705698

[B61] Wendell S. G. Baffi C. Holguin F. (2014). Fatty acids, inflammation, and asthma. J. Allergy Clin. Immunol. 133, 1255–1264. doi: 10.1016/j.jaci.2013.12.1087 24613565 PMC4417548

[B62] Wu M. Z. Chandramouli C. Wong P. F. Chan Y. H. Li H. L. Yu S. Y.. (2022). Risk of sepsis and pneumonia in patients initiated on SGLT2 inhibitors and DPP-4 inhibitors. Diabetes Metab. 48, 101367. doi: 10.1016/j.diabet.2022.101367 35753654

[B63] Wu F. Huang Y. Hu J. Shao Z. (2020). Mendelian randomization study of inflammatory bowel disease and bone mineral density. BMC Med. 18, 312. doi: 10.1186/s12916-020-01778-5 33167994 PMC7654011

[B64] Xu M. Zheng J. Hou T. Lin H. Wang T. Wang S.. (2022). SGLT2 inhibition, choline metabolites, and cardiometabolic diseases: A mediation mendelian randomization study. Diabetes Care 45, 2718–2728. doi: 10.2337/dc22-0323 36161993 PMC9862376

[B65] Ye S. Wang L. Li S. Ding Q. Wang Y. Wan X.. (2022). The correlation between dysfunctional intestinal flora and pathology feature of patients with pulmonary tuberculosis. Front. Cell Infect. Microbiol. 12. doi: 10.3389/fcimb.2022.1090889 PMC9811264 36619765

[B66] Yi W. J. Han Y. S. Wei L. L. Shi L. Y. Huang H. Jiang T. T.. (2019). l-Histidine, arachidonic acid, biliverdin, and l-cysteine-glutathione disulfide as potential biomarkers for cured pulmonary tuberculosis. BioMed. Pharmacother. 116, 108980. doi: 10.1016/j.biopha.2019.108980 31125821

[B67] Yu G. Xu C. Zhang D. Ju F. Ni Y. (2022). MetOrigin: Discriminating the origins of microbial metabolites for integrative analysis of the gut microbiome and metabolome. iMeta 1, e10. doi: 10.1002/imt2.10 38867728 PMC10989983

[B68] Zannad F. Ferreira J. P. Pocock S. J. Anker S. D. Butler J. Filippatos G.. (2020). SGLT2 inhibitors in patients with heart failure with reduced ejection fraction: a meta-analysis of the EMPEROR-Reduced and DAPA-HF trials. Lancet 396, 819–829. doi: 10.1016/s0140-6736(20)31824-9 32877652

[B69] Zhang T. Cao Y. Zhao J. Yao J. Liu G. (2023). Assessing the causal effect of genetically predicted metabolites and metabolic pathways on stroke. J. Transl. Med. 21, 822. doi: 10.1186/s12967-023-04677-4 37978512 PMC10655369

[B70] Zhou M. Wang H. Zeng X. Yin P. Zhu J. Chen W.. (2019). Mortality, morbidity, and risk factors in China and its provinces 1990-2017: a systematic analysis for the Global Burden of Disease Study 2017. Lancet 394, 1145–1158. doi: 10.1016/s0140-6736(19)30427-1 31248666 PMC6891889

[B71] Zimmermann P. Messina N. Mohn W. W. Finlay B. B. Curtis N. (2019). Association between the intestinal microbiota and allergic sensitization, eczema, and asthma: A systematic review. J. Allergy Clin. Immunol. 143, 467–485. doi: 10.1016/j.jaci.2018.09.025 30600099

